# Obesity induces phenotypic switching of gastric smooth muscle cells through the activation of the PPARD/PDK4/ANGPTL4 pathway

**DOI:** 10.1186/s12929-025-01163-5

**Published:** 2025-07-12

**Authors:** Sanaa Dekkar, Kamilia Mahloul, Amandine Falco, Karidia Konate, Romane Pisteur, Sarah Maurel, Laurent Maïmoun, Norbert Chauvet, Prisca Boisguérin, David Nocca, Ariane Sultan, Florian Pallot, Guillaume Walther, Nicolas Cenac, Cyril Breuker, Sandrine Faure, Pascal de Santa Barbara

**Affiliations:** 1 PHYMEDEXP, University of Montpellier, INSERM, CNRS, Montpellier, France; 2 IRSD, INSERM, INRAe, ENVT, UPS, University of Toulouse, Toulouse, France; 3 Department of Nuclear Medicine, University Hospital of Montpellier, Montpellier, France; 4 Department of Digestive Surgery, University Hospital of Montpellier, Montpellier, France; 5 Department of Nutrition-Diabetes, University Hospital of Montpellier, Montpellier, France; 6 LAPEC, University of Avignon, Avignon, France; 7 Department of Clinical Pharmacy, University Hospital of Montpellier, Montpellier, France

**Keywords:** Smooth muscle, Obesity, Stomach, Immaturity, PDK4/ANGPTL4/PPARD pathway

## Abstract

**Background:**

Clinical research has identified stomach dysmotility as a common feature of obesity. However, the specific mechanisms driving gastric emptying dysfunction in patients with obesity remain largely unknown. In this study, we investigated potential mechanisms by focusing on the homeostasis of gastric smooth muscle.

**Methods:**

An obese mouse model was established using a high-fat diet (HFD). Immunofluorescence analysis and Western blotting were employed to assess smooth muscle status using stage-specific markers. An in vitro culture model of differentiated human gastric smooth muscle cells (SMCs) was treated with lipids, siRNA-peptide-based nanoparticles and pharmaceutical compounds. Global lipidomic and RNA sequencing analyses were performed. The findings were evaluated in patients with obesity, using gastric samples from individuals who underwent sleeve gastrectomy, to evaluate their clinical relevance.

**Results:**

The smooth muscle layers in gastric tissue from both mice fed on a HFD as well as patients with obesity exhibited altered differentiation status. Treatment of differentiated human gastric SMCs with lipids phenocopies these alterations and is associated with increased expression of *PDK4* and *ANGPTL4*. Inhibition of PDK4 or ANGPTL4 upregulation prevents these lipid-induced modifications. PPARD activation stimulates *PDK4* and *ANGPTL4* upregulation, leading to SMC dedifferentiation. Notably, *PDK4* and *ANGPTL4* levels correlate with immaturity and alteration of gastric smooth muscle in patients with obesity.

**Conclusions:**

Obesity triggers a phenotypic change in gastric SMCs, driven by the activation of the PPARD/PDK4/ANGPTL4 pathway. These mechanistic insights offer potential biomarkers for diagnosing stomach dysmotility in patients with obesity.

**Supplementary Information:**

The online version contains supplementary material available at 10.1186/s12929-025-01163-5.

## Background

Obesity is a prevalent chronic condition affecting approximately 13% of the adult population in the world, and constitutes a major and widespread global public health issue [1]. It results from mechanisms such as an imbalance between energy intake and expenditure, eating disorders and is strongly associated with various medical conditions, including type 2 diabetes, and cardiovascular diseases. There is also strong evidence for an association of obesity with several cancer types (endometrial, postmenopausal breast, prostate, and renal) [2]. Individuals with obesity often experience gastrointestinal (GI) issues including gastroesophageal reflux, nonalcoholic fatty liver disease, and an elevated risk of colorectal and esophageal adenocarcinoma. Furthermore, functional GI disorders like dyspepsia, inflammatory bowel syndrome, constipation and diarrhea are frequently observed in these patients [3–5]. Despite their prevalence, GI complications associated with obesity remains insufficiently explored in both clinical practice and research.

Conflicting findings have been reported in studies investigating the impact of obesity on GI motility, likely due to the inherent regionalization and segmentation of the GI tract along the rostro-caudal axis, which complicates the assessment of obesity's effects on GI function [6, 7]. In line with the stomach's role—alongside the brain—in regulating food intake, gastric emptying and satiety mechanisms, studies conducted on genetically and diet-induced obese mice have demonstrated significant alterations in gene expression in the stomach rather than in the small intestine or colon [7, 8]. Several studies reported accelerated gastric emptying in patients with obesity, potentially leading to disrupted satiety signaling, and increased food consumption. This could contribute to the pathogenesis of obesity and its related complications [9–12]. Supporting this hypothesis, recent findings have associated faster gastric emptying with significant weight gain in younger adults [13].

Gastric emptying is a complex functional mechanism that requires the generation and conduction of regular depolarizing potential (slow waves) in the smooth muscle, events initiated by the interstitial cells of Cajal (ICC) which are electrically coupled to the smooth muscle cells (SMC) [14–16]. Interestingly, the stomach of early-induced obese mice shows increased proliferation and density of ICCs, contributing to the accelerated gastric emptying observed in these obese mice [17]. However, the impact of obesity on the gastric smooth muscle has been undervalued, despite disturbances in its activity potentially leading to motility issues [18–20]. This is mainly due to an incomplete understanding of GI smooth muscle physiology.

Digestive SMCs originate from LIX1-positive mesenchymal progenitors through a two-step process [21, 22]. These progenitors first undergo a determination program characterized by the expression of MYOCARDIN, a cofactor of the transcription factor SRF (Serum Response Factor), which regulates the expression of smooth muscle actin proteins, including gamma (gSMA) and alpha (aSMA) smooth muscle actins [23]. Following this, determined SMCs enter a more differentiated state, marked by cell elongation and the expression of CALPONIN1 and SM22, two actin-binding proteins involved in smooth muscle contractility [19]. As SMCs differentiate, they acquire the capacity to generate force and contract, facilitated by the presence and organization of smooth muscle actin and myosin proteins. While skeletal muscle contains satellite cells that respond to growth and injury, a comparable reservoir of cells presenting pluripotency capacities has not been identified in smooth muscles. Instead, SMCs possess the capacity to dedifferentiate upon stimulation, revert to a synthetic mesenchymal phenotype, and re-enter the cell division cycle to proliferate [18, 19]. This physiological process, named phenotypic switching, is crucial during the growth of the pediatric intestine smooth muscle [24]. However, an imbalance in plasticity favoring the mesenchymal progenitor state is thought to contribute to numerous diseases, including primary visceral myopathy, inflammatory bowel disease, diabetes, and metabolic disorders [20, 25], ultimately leading to impaired GI motility [18, 19].

In this study, we thoroughly examined gastric homeostasis in the context of obesity, with a particular focus on muscular changes. Analysis of tissues from diet-induced obesity mouse model revealed alterations in SMC differentiation. Using a novel in vitro model of differentiated human gastric SMCs, we demonstrated that lipids act directly on SMC and identified the PPARD/PDK4/ANGPTL4 pathway as a key player in SMC plasticity. Evaluation of this pathway in patients with obesity highlighted that gastric SMCs in this population undergo phenotypic switching.

## Methods

### Human tissues and evaluation

The study involved collecting gastric wall samples from two patient groups. The first group included 15 patients with obesity (body mass index (BMI) ≥ 35 kg/m^2^) who underwent sleeve gastrectomy between September and December 2018 (Additional file 1: Table S1). The second group comprised 4 lean control patients with a BMI below 30 kg/m^2^ who underwent surgeries for gastric or esophageal epithelial tumors in January 2019 (Additional file 1: Table S2). Participants were included regardless of their weight or diabetic status and their biological data were collected. Samples were collected in accordance with the ethical guidelines of Montpellier University Hospital (France), with informed consent obtained from each patient, permitting the use of their tissue samples for research purposes. Human gastric wall samples were collected and the corpus of the stomach were processed for different types of analyses. For histology and immunohistochemistry, samples were fixed in 4% paraformaldehyde in PBS (Corning), dehydrated with ethanol, and embedded in paraffin following standard protocols [26]. For molecular analyses, a portion of the samples underwent microdissection to isolate gastric smooth muscle fibers, which were then rapidly frozen in liquid nitrogen and stored at −80 °C. These frozen samples were later used for RT-qPCR and Western Blot analyses to assess gene expression and protein levels.

### High-Fat diet mice treatment and evaluation

Male C57BL/6 J mice (10–11 weeks old) from Janvier Laboratories (Saint-Berthevin Cedex, France) were individually housed under controlled conditions with a 12-h light–dark cycle and had unrestricted access to food and water. The study adhered to the European Parliament Directive 2010/63/EU and was approved by the local ethics committee of Marseille (Approval Number: 2020050612125728). Male mice were randomly divided into two groups (n = 7 per group): one group received a normal chow diet (Control) and the other a High-Fat Diet (HFD) containing 60% fat (230-HFD). After 12 weeks of the diet, mice were assessed for changes in metabolism and after for biochemistry. Then, the animals were anesthetized with an intra-peritoneal injection of Dolethal (182.2 mg pentobarbital/kg body weight, Vetoquinol) and their stomachs were collected. These samples were either fixed in 10% formaldehyde for histological analysis or frozen in liquid nitrogen and stored at − 80 °C for biochemical assays.

### Mouse metabolism evaluation

After 12 weeks of diet, mice from both groups underwent oral glucose tolerance tests (OGTT), and insulin tolerance tests (ITT) [27]. The OGTT was conducted at the end of the 12th week on each groups. Following an overnight fast, fasting blood glucose levels were measured using blood collected via tail clip (Caresens^®^ N, DinnoSanteTM). The mice received a glucose solution by oral gavage (1.5 g/kg), and blood glucose levels were measured at 10, 20, 30, 45, 60, 90, and 120 min post-administration During the same week, the other half of the group (n = 7 per group) underwent an insulin tolerance test (ITT). Following a six-hour fasting period, baseline blood glucose levels were measured. Mice were then administered an intraperitoneal injection of insulin (1 UI/kg), and blood glucose levels were subsequently measured at 10, 20, 30, 45, 60, 90, and 120 min post-injection.

### Human gastric smooth muscle cell and treatment

Human gastric SMCs (Innoprot Innovative, Spain) were cultured on collagen I-coated dishes (Corning) in Dulbecco’s Modified Eagle’s Medium (DMEM) with 10% fetal bovine serum (Sigma) and 1% penicillin/streptomycin [22]. Differentiation was induced over 14 days, with lipid and pharmacological treatments beginning after differentiation. For lipid treatments, SMCs were exposed to a 2% lipid mixture (Sigma) supplemented with BSA-complexed long-chain fatty acids (30 µM palmitic acid, and 30 µM oleic acid), or a 60 µM BSA (fatty acid-free preparation of BSA) as control for 3, 7 and 14 days. In the reversibility assays, cells were washed after 7 days of lipid treatments and maintain in culture for an additional 7 days in medium with 60 µM BSA. In the PPARD antagonist experiments, reversible (GSK0660) and covalent (GSK3787) antagonist were used. For GSK0660, differentiated SMCs were serum-starved for 24 h and treated with 50 µM GSK0660 (reversible antagonist) or 0.1% DMSO for 24 h, followed by exposure to the lipid treatment with additional GSK0660 every 24 h for 72 h. For GSK3787, differentiated SMCs were treated with 5 µM GSK3787 or 0.1% DMSO in the presence of lipid treatment in a serum free medium for 72 h. For PPARD agonist experiments, differentiated SMCs were treated with 1 µM GW501516 or 0.1% DMSO for 24 or 72 h. To inhibit lipid-induced *PDK4* and *ANGPTL4* expression, differentiated SMCs were treated with WRAP5-siRNA nanoparticles targeting *PDK4*, *ANGPTL4*, or a control siRNA for 2 h, then cultured in a lipid treatment for 3 days. For Actinomycin D (ActD) treatments, SMCs were first exposed to lipid treatment or to 60 µM BSA (used as a control) for 3 days, washed with serum-free medium, and then treated with 1 µg/ml ActD for 30 min, 1, 2, 4, and 6 h. Analysis included immunofluorescence, Western blotting, quantitative RT-PCR, and assessments of cell viability and toxicity. Gastric SMCs were routinely screened for mycoplasm contamination using the MycoAlert^®^ Detection Kit (Lonza).

### RNA-Seq library preparation, sequencing and analysis

RNA libraries were prepared from SMCs treated with or without a lipid mixture for 3 and 7 days (n = 3 per condition) using the TruSeq Stranded mRNA Library Prep Kit (Illumina, ref. RS-122-2101), following the manufacturer’s instructions (MGX, Biocampus, France). The libraries were validated with a Fragment Analyzer (Agilent) and quantified using the KAPA Library Quantification Kit (Roche, ref. KK4824). Libraries were then pooled in equimolar ratios and sequenced on a HiSeq2500 platform with a single-read protocol (50 nt, 1.5 lane flowcell). Image analysis and base calling were performed with Illumina HiSeq Control Software and the Real-Time Analysis component. Demultiplexing was done using Illumina’s bcl2fastq 2.18 software. The quality of raw sequence data was assessed using FastQC (Babraham Institute) and SAV (Sequencing Analysis Viewer), with potential contaminants identified via FastQ Screen (Babraham Institute). RNA-seq reads were aligned to the human genome (UCSC Hg38) using TopHat and Bowtie, with gene model annotations from the UCSC database (as of January 14, 2019). Alignments with more than three mismatches were excluded. Read counts for each gene were obtained using HTSeq-count 0.9.0.20 in union mode. Genes with fewer than 15 reads across all samples were filtered out before statistical analysis. Read counts were normalized with the Relative Log Expression (RLE) method in the Bioconductor package EdgeR. Differentially expressed genes were identified using EdgeR and DESeq2, with P-values adjusted for multiple testing using the Benjamini–Hochberg False Discovery Rate (FDR) method.

### Lipidomic analysis

The lipid composition of the treated SMC cultures was analyzed using an Agilent 1290 UPLC system coupled to a G6460 triple quadripole spectrometer (Agilent Technologies) for the ceramides and the sphingomyelins, and by gas–liquid chromatography on a Clarus 600 Perkin Elmer system using a Famewax RESTEK fused silica capillary columns for total fatty acid (fatty acid methyl esters), as previously described [28]. Relative concentration (pg/mg of proteins) of fatty acids, ceramides and sphingomyelins are presented in Additional file 1: Tables S3, S4.

### Nanoparticle formulation and characterization

The WRAP5 peptide was synthesized at the SynBio3 platform (IBMM, Montpellier) using the Fmoc strategy, following established methods [29]. Crude peptide products were purified in-house and analyzed by HPLC/MS to ensure purity greater than 95%. WRAP5 was dissolved in water (Sigma-Aldrich) to a final concentration of 400 µM (stock solution, stored at 4 °C). *PDK4*- and *ANGPTL4*-siRNAs (Additional file 1: Table S5) and a control siRNA (si-*Neg.*, Ref.: SR-CL000-005) were purchased from Eurogentec (France) and dissolved in RNase-free water to a final concentration of 20 µM (stock solution, stored at − 20 °C). For siRNA-loaded WRAP5 nanoparticle preparation, WRAP5 and siRNA were mixed at a 20:1 molar ratio in water with 5% glucose at room temperature. The nanoparticles were characterized by Dynamic Light Scattering (DLS) using a Zetasizer NanoZS (Malvern) to assess their mean size (Z-average) and distribution homogeneity (PdI). They were then stored at 4 °C for 24 h before use on human gastric SMCs.

### Cell viability and cytotoxicity measurement

To assess the effects of lipid treatment (2% lipid mixture (Sigma; Ref. L0288) with BSA-complexed long-chain fatty acids, 30 µM palmitic acid conjugated to BSA (Bertin; Ref. 29,558), and 30 µM oleic acid conjugated to BSA (Bertin; Ref. 29,557)), or a 60 µM BSA control (Bertin; Ref. 29556) and WRAP5-siRNA nanoparticles on human gastric SMCs (Innoprot Innovative, Spain) [22], cell viability and cytotoxicity were evaluated respectively using flow cytometry and LDH assays. For viability, cells were treated with Trypsin, resuspended in Muse^®^ Count & Viability Reagent, and analyzed with a Muse^®^ Cell Analyzer. Cytotoxicity was measured using the LDH Cytotoxicity Detection KitPlus: supernatant samples were tested, with Triton X-100 as a positive control and a non-treated well as a negative control. Absorbance was read at 490 nm, and relative toxicity was calculated based on the formula: [(experimental value—non-treated value)/(Triton value—non-treated value)] × 100.

### Transmission electron microscopy and immunofluorescence

For transmission electron microscopy (TEM), human gastric SMCs treated with either lipids or BSA as control for 7 days were fixed in 2.5% glutaraldehyde in PHEM buffer (pH 7.2) at room temperature for 1 h. Cells were then post-fixed in 0.5% osmium tetroxide in the dark for 2 h. After dehydration through graded ethanol solutions (30% to 100%), SMCs were embedded in EmBed 812 resin using an Automated Microwave Tissue Processor for Electron Microscopy (Leica EM AMW). Ultra-thin Sects. (70 nm) were cut with a Leica-Reichert Ultracut E microtome, stained with 1.5% uranyl acetate and lead citrate, and examined with a Tecnai F20 TEM at 200 kV at CoMET MRI facilities, INM, Montpellier, France. For immunofluorescence, human gastric SMCs were seeded on Collagen Type I-coated coverslips (50 µg/mL per coverslips), allowed to differentiate, and treated or not with lipid mixture. Cells were fixed with 4% paraformaldehyde in PBS, permeabilized with 0.1% Triton X-100, and blocked with 10% goat serum in PBS-tween 0.1%. They were then incubated with primary and secondary antibodies or stained with HCS CellMask Red 588/612 and Bodipy 493/503 for neutral lipids (Fisher Scientific, France, Ref. 11540326)[30]. Antibodies are listed in Additional file 1: Table S6. Nuclei were labeled with Hoechst. All samples were mounted in home-made Mowiol.

### RNA isolation and qPCR analysis

Total RNA was extracted from cell cultures with the RNeasy^®^ Mini Kit (Qiagen) and from tissues using a Polytron homogenizer and Trizol lysis buffer (Invitrogen, Ref.: AM9738), according to the manufacturers'protocols. The RNA was then reverse transcribed into cDNA using the Verso cDNA Synthesis Kit (Thermo Scientific). Quantitative PCR (qPCR) was conducted with LightCycler technology (Roche Diagnostics). PCR primers were designed using the National Library of Medicine database (Additional file 1: Table S7). Gene expression levels were quantified with LightCycler analysis software (version 3.5) relative to standard curves. The results are presented as mean gene expression levels normalized to reference genes HMBS, YWHAZ and RPLPO calculated using the 2^-ΔΔCt method.

### Western blots

Human gastric SMC cultures and patient-derived smooth muscle fibers were lysed using RIPA buffer, which consisted of 150 mM NaCl, 1% Triton X-100, 1% SDS, 50 mM Tris (pH 8), and a cOmplete EDTA-free protease inhibitor cocktail (Roche). Mouse stomach tissues were homogenized in a lysis buffer containing 50 mM Tris–HCl (pH 8), 2 mM EGTA, 1 mM DTT, 0.2% NP40, 0.02% SDS, and the same protease inhibitor cocktail. The lysates were incubated on ice for 1 h, then centrifuged to collect the proteins. Protein concentrations were measured using DC^™^ protein assay kit (Bio-Rad) according to the manufacturer’s instructions. For protein analysis, 10 µg of protein from each sample were separated by 12% SDS-PAGE, transferred to nitrocellulose membranes, and subjected to Western blotting. The membranes were incubated with primary antibodies, detected with infrared-labeled secondary antibodies, and imaged using the Odyssey infrared imaging system (LI-COR Biosystems). The antibodies used are listed in Additional file 1: Table S6.

### Tissue immunohistochemistry and immunofluorescence

The paraffin-embedded block was sectioned into 10 μm thick slices. After deparaffinization and antigen retrieval (using 0.01 M citrate buffer, pH 6.0, at 96 °C for 30 min), slides were immunostained following standard procedures [26]. Tissue sections were incubated overnight at 4 °C with primary antibodies, then detected with biotinylated secondary antibodies. Detection was amplified using the Streptavidin/Biotin kit (Vector) and visualized with 3,3’ diaminobenzidine (Sigma, France), followed by counterstaining with hematoxylin. For immunofluorescence staining, detection was done with corresponding Alexa secondary antibodies in the presence of Hoechst 33,342 (Invitrogen) for nuclei labelling. All sections were rinsed and mounted using Mounting Medium (DAKO). Images were captured with a Nikon Multizoom AZ100 stereomicroscope and a Carl Zeiss AxioImager microscope. Antibodies used are listed in Additional file 1: Table S6.

### Statistical analysis

Data are presented as means ± standard error of the mean (SEM). Statistical analyses were performed using GraphPad Prism 9.0 software (GraphPad, San Diego, CA) with the two-tailed Mann–Whitney test. For multiple comparisons within groups, the Kruskal–Wallis test followed by Dunn’s multiple comparisons test was employed. Pearson’s correlation test was used for correlating gene and protein expression. Simple linear regression test and mixed effect analysis for multiple comparisons were used for Actinomycin D chase experiments. Statistical significance was set at P < 0.05. Details of the statistical tests used are provided in the legend of each Figure.

## Results

### Obesity is associated with altered gastric smooth muscle differentiation in mice

**Fig. 1:**
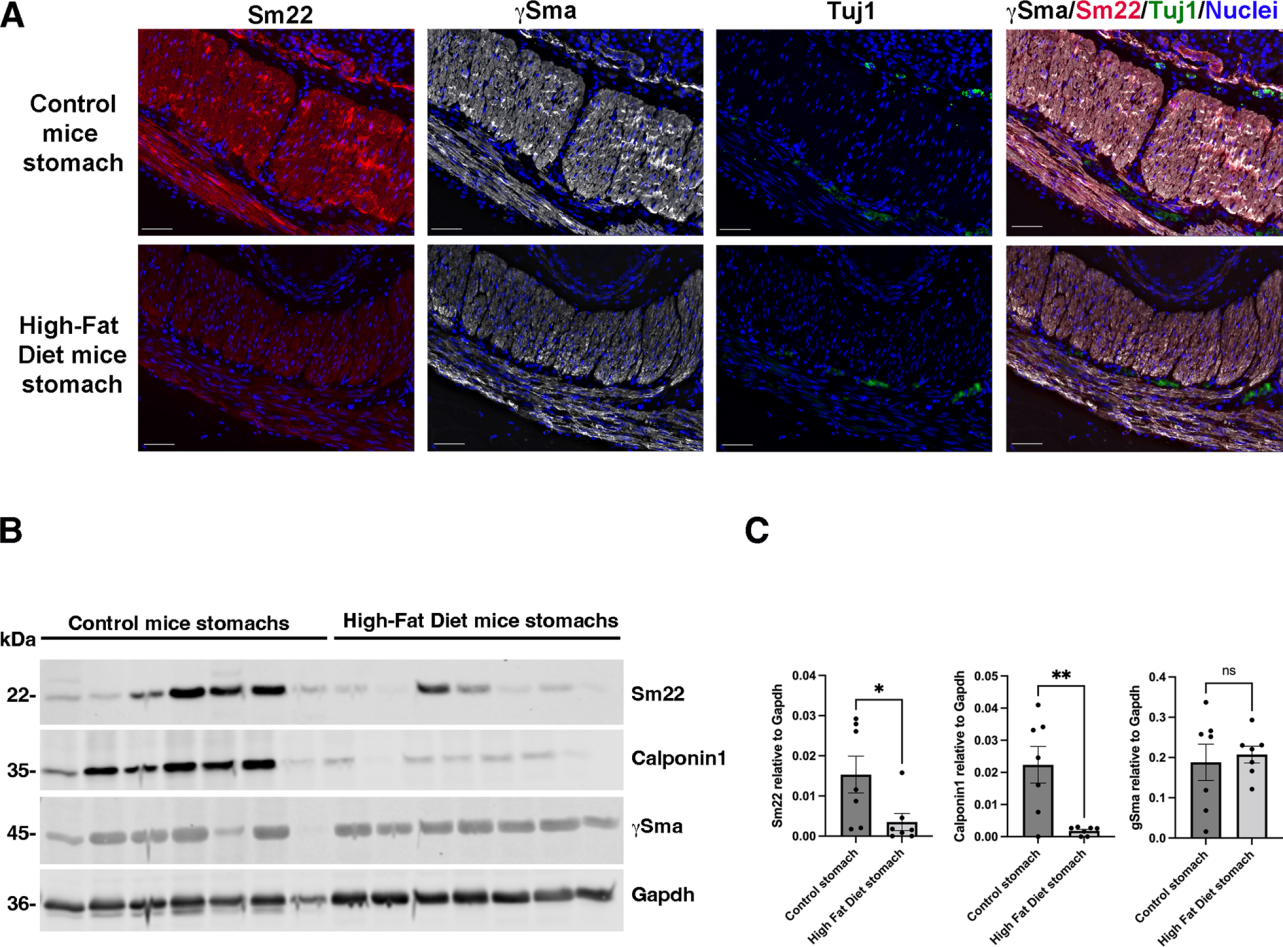
Evaluation of the differentiation status of gastric smooth muscle in HFD-induced obese mice. **A** Representative immunofluorescence staining of smooth muscle (Sm22 and gSma) and enteric neuron (Tuj1) markers in control (upper panels) and HFD (lower panels) mouse stomach section. Scale bars: 50 mm. Immunofluorescence analysis highlighted a reduced level of Sm22 in the stomach musculature of HFD mice compared to controls, whereas the expression level of gSma remained unchanged. No change in smooth muscle layer organization was observed. We also found the presence of myenteric neurons (Tuj1 positive clusters) in both conditions. **B** Representative Western-blot image of whole stomach extracts from adult mice fed with a HFD for 12 weeks (n = 7), and from controls (n = 7) probed with antibodies directed against specific smooth muscle proteins (Sm22, Calponin1, and gSma) and against Gapdh as loading control. **C** Quantification of the Western-blot (**B**) comparing extracts from HFD stomach extracts from controls. Data are presented as the mean ± SEM (two-tailed Mann–Whitney test; *P < 0.05; **P < 0.01). The expression of Sm22 and Calponin1 was significantly lower in HFD compared to control condition (**B**, **C**)

To explore potential alterations linked to obesity, we analyzed the gastric musculature in a mouse model of induced obesity. Male adult mice were subjected to a High-Fat Diet (HFD) for 12 weeks, a period known to induce various obesity-related metabolic changes [27]. This dietary regimen effectively induced obesity, as evidenced by the appearance of multiple metabolic changes commonly associated with the condition (Additional file 2: Fig. 1). Using immunofluorescence approaches, we first evaluated the differentiation status of gastric smooth muscle cells (SMCs) using specific SMC markers, respectively gSma, a marker defining SMC determination and identity and Sm22, a marker of SMC differentiation status. We observed a reduced level of Sm22 in the gastric musculature of HFD mice compared to controls, whereas the expression level of gSma remained unchanged (Fig. 1A). To verify the presence and integrity of enteric neurons in the stomachs of HFD-treated mice, we used enteric neuronal marker (Tuj1) marker and observed that Tuj1 signal across experimental groups is preserved supporting that the enteric neuronal network remained largely intact (Fig. 1A). Western blot analysis revealed a significant reduction in Sm22 and Calponin1 levels (markers of SMC differentiation status) in gastric extracts prepared from obese mice compared to controls (Fig. 1B, C). gSma levels remained constant indicating a specific deregulation of the differentiation status of the SMCs (Fig. 1B, C). These findings suggest a link between early induced obesity and alterations in the differentiation status of gastric smooth muscle.

### Lipid treatment altered gastric smooth muscle cell differentiation

**Fig. 2:**
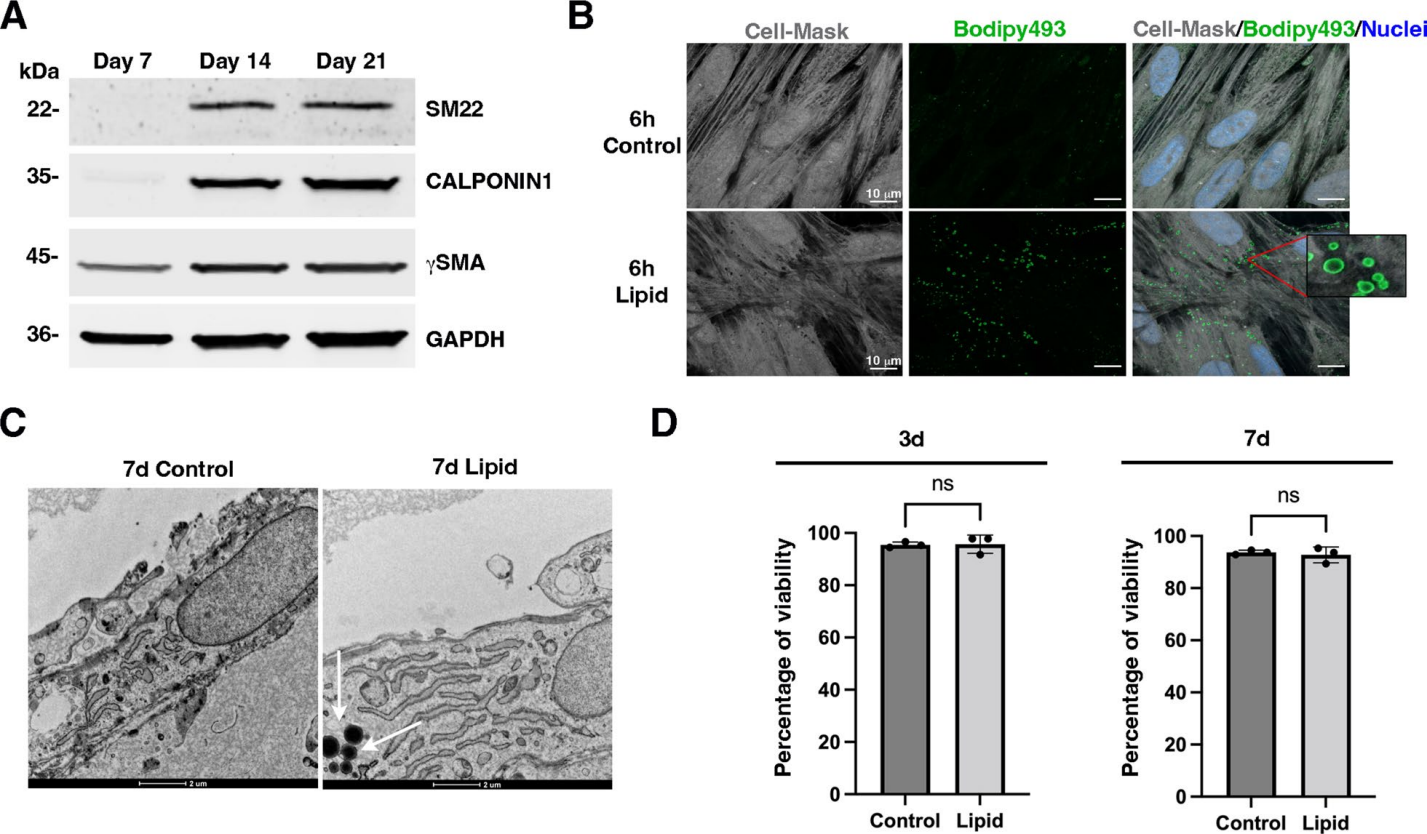
Development and characterization of human gastric differentiated smooth muscle cells. Human gastric SMCs were cultured on Collagen Type I plates over time to promote their differentiation. **A** Representative Western blot of human gastric SMC extracts from 7-, 14- and 21-days of culture probed with antibodies directed against specific smooth muscle proteins (SM22, CALPONIN1, and gSMA) and against GAPDH as loading control. **B** 14-day human gastric SMCs were incubated with lipid treatment for 6 h, followed by staining with Bodipy493/503 and Cell-Mask Red stain 588/612 for 30 min, and nuclei staining with Hoechst. Scale bars: 10 mm. Numerous lipid structures within lipid-treated SMCs were detected with Bodipy staining (see zoomed insert) by confocal microscopy. **C** 14-day human gastric SMC cultures with or without lipid treatment for 7 days were analyzed by transmission electron microscopy. White arrows indicate the presence of lipid droplets observed only in the lipid-treated conditions. Scale bars: 2 mm. **D** Cell viability of human gastric SMC cultures after 14 days with or without lipid treatment for an additional 3 or 7 days was evaluated by flow cytometry following differential DNA-binding dye staining. No significant impact on cell viability was observed after 3 days or 7 days of lipid treatment. Data are presented as the mean ± SEM and Mann–Whitney was applied (ns > 0.05)

Obesity has been shown in the small intestine to disrupt epithelium homeostasis and alter the composition of the enteric nervous system [31, 32], which may contribute to changes in smooth muscle via epithelial-mesenchymal or neuro-mesenchymal interactions [19, 33]. To explore whether dietary constituents of the HFD directly influence the gastric smooth muscle, we first developed an in vitro culture model of differentiated human gastric SMCs. In vitro studies on SMCs are challenging due to their tendency to spontaneously shift toward synthetic and undifferentiated states [18–20, 22]. Research on uterine SMCs has demonstrated that low-density cultures reduce contractile marker expression, while confluence enhances differentiation and reduces proliferative capacity [34]. In our study, culturing gSMA-positive human gastric SMCs to confluence over 14 days led to the expression of SM22 and CALPONIN1, designating them as differentiated SMCs (Fig. 2A).

**Fig. 3:**
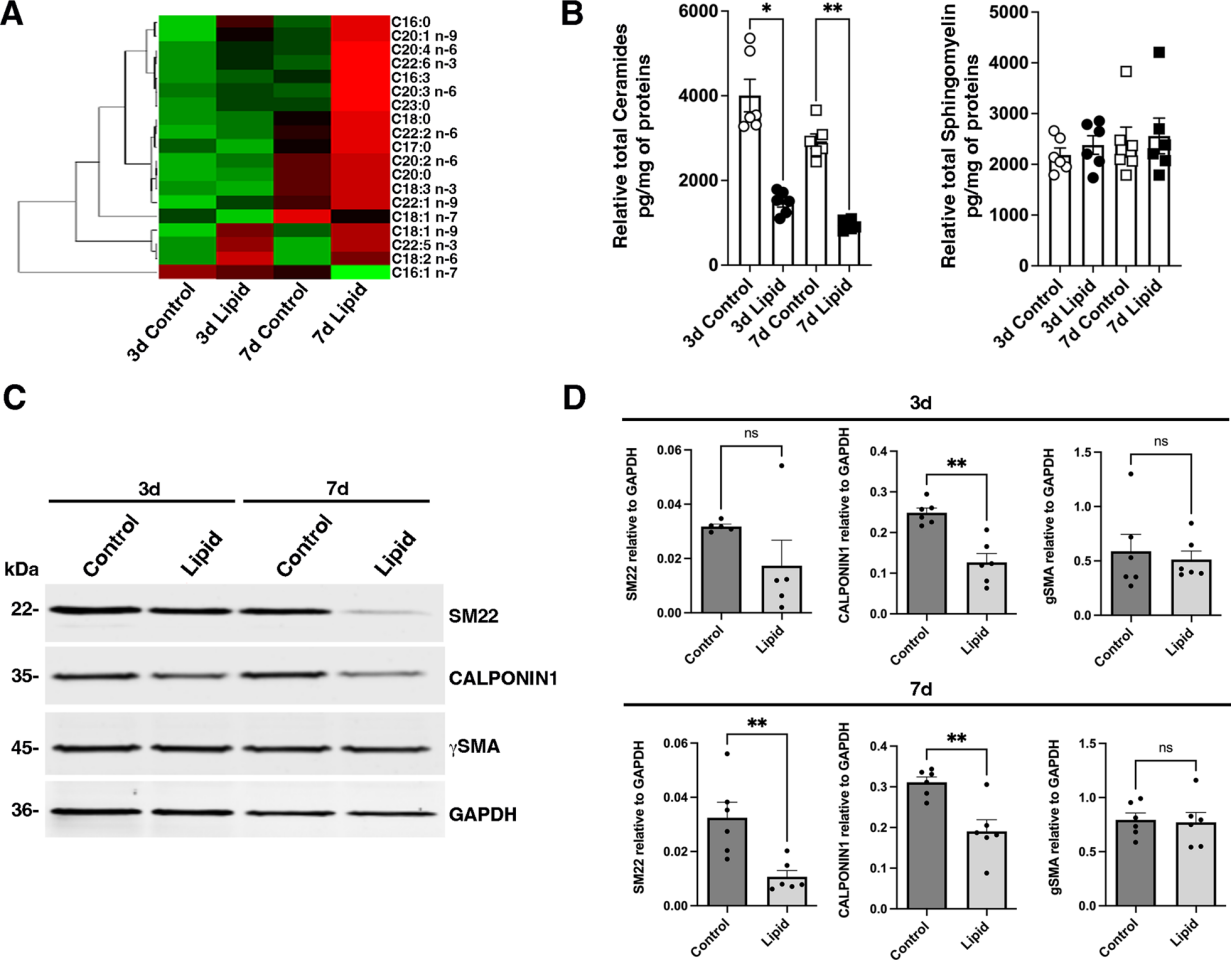
Impact of lipid treatment on human gastric smooth muscle cells. **A** Heat map of fatty acids quantified in SMCs with or without lipid treatment for an additional 3 and 7 days. The data is shown in a matrix format: each row represents a single lipid, and each column represents a SMC treatment: control day 3, lipid day 3, control day 7, and lipid day 7. Each color patch represents the normalized quantity of lipid (row) in treated SMC (column), with a gradient from bright green (lowest) to bright red (highest). The pattern and length of the branches in the left dendrogram reflect the relatedness of the lipids. All data were normalized to the quantity of protein (pg/mg of proteins). **B** Levels of total ceramides (left panel) and total sphingomyelins (right panel) quantified in SMCs with or without lipid treatment for an additional 3 and 7 days. All data were normalized to the quantity of protein (pg/mg of proteins). Values are the mean ± SEM of n = 6 samples. *P < 0.05 and **P < 0.01 (non-parametric Kruskall-Wallis test with Dunn’s multiple comparison test). **C** Representative Western blot of human gastric SMC extracts after 14 days of culture with or without lipid treatment for an additional 3 or 7 days probed with antibodies directed against specific smooth muscle proteins (SM22, CALPONIN1, and gSMA) and against GAPDH as loading control. **D** Quantification of Western blot assays comparing extracts from lipid-treated SMCs to extracts from control SMCs. Data are presented as the mean ± SEM and two-tailed Mann–Whitney test was applied (*P < 0.05; ns > 0.05). CALPONIN1 expression was significantly reduced after 3 days, while SM22 was significantly lower only after 7 days of treatment (**C**, **D**)

Lipid components of the HFD, including long-chain fatty acids such as palmitic acid and oleic acid, have been shown to be sufficient to replicate the in vivo HFD-induced increase in intestinal epithelial progenitors [31]. We directly exposed differentiated SMCs to these lipids. We observed the rapid internalization of the lipids in differentiated SMCs, as evidenced by Bodipy detection through confocal microscopy after 6 h (Fig. 2B). Further intracellular accumulation of lipids in lipid droplets was confirmed, via transmission electron microscopy, 7 days post-treatment (Fig. 2C, white arrows). Importantly, SMC viability remained unaffected by lipid treatment after both 3 and 7 days of exposure (Fig. 2D). Liquid chromatography-tandem mass spectrometry lipidomic analysis of the SMC membrane revealed an increase in fatty acids, primarily polyunsaturated fatty acids, at both 3 and 7 days of treatment (Fig. 3A; Additional file 1: Table S3). This was accompanied by a decrease in total ceramides, with no changes in total sphingomyelins, suggesting a pre-metabolic condition (Fig. 3B; Additional file 1: Table S4). Next, we assessed the impact of lipid treatment on the expression of SMC-specific markers. Notably, 3 days of lipid exposure led to a significant reduction in CALPONIN1 protein levels. Both SM22 and CALPONIN1 levels were further reduced after 7 days of treatment, while γSMA expression remained unchanged (Figs. 3C, D). These findings highlight the direct role of lipids in promoting SMC dedifferentiation.

### PDK4 and ANGPTL4 are essential in lipid-induced dedifferentiation of human gastric SMCs

**Fig. 4:**
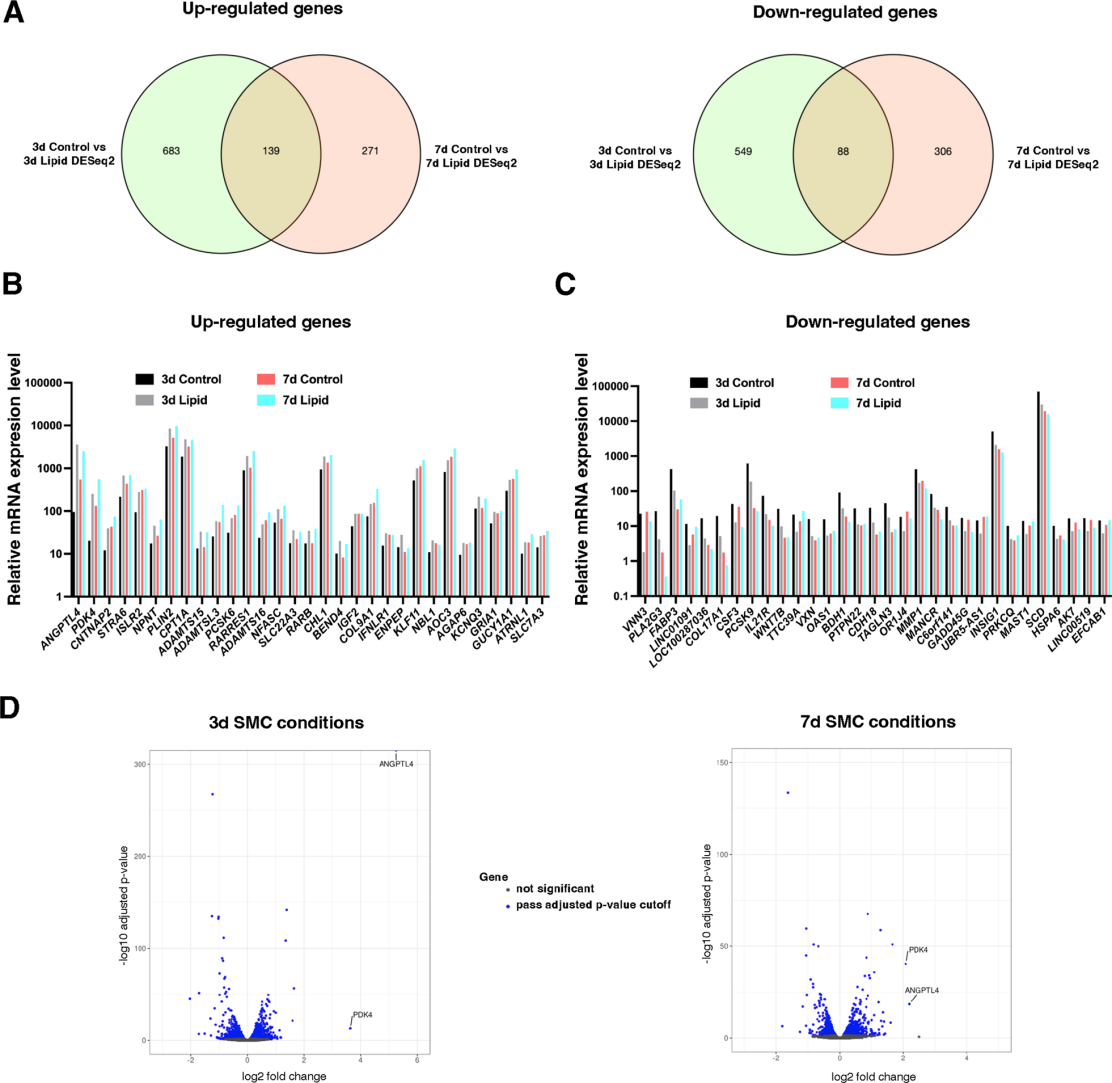
Identification of the molecular mechanism induced by lipid treatment in human gastric SMC. **A** Venn diagram depicting the number of genes up-regulated (left panel) and down-regulated (right panel) identified at 3 days and 7 days post-lipid treatment using a significance cutoff of p < 0.01. For both times, 139 genes were up-regulated, and 88 genes were down-regulated in response to lipid treatment. Relative mRNA expression of upregulated (**B**) and downregulated (**C**) genes identified by RNA sequencing using a significance cutoff of p < 0.01 in human gastric SMC cultures with or without lipid treatment for 3 and 7 days. Average of n = 3 (**B**, **C**). **D** Volcano plot showing differentially expressed genes between human gastric SMCs treated with lipid for 3 days (left panel) or 7 days (right panel) compared to untreated SMCs. Blue dots indicate genes with a significance cutoff of p < 0.01. Among them, upregulation of *PDK4* and *ANGPTL4* levels are found in both time conditions

**Fig. 5:**
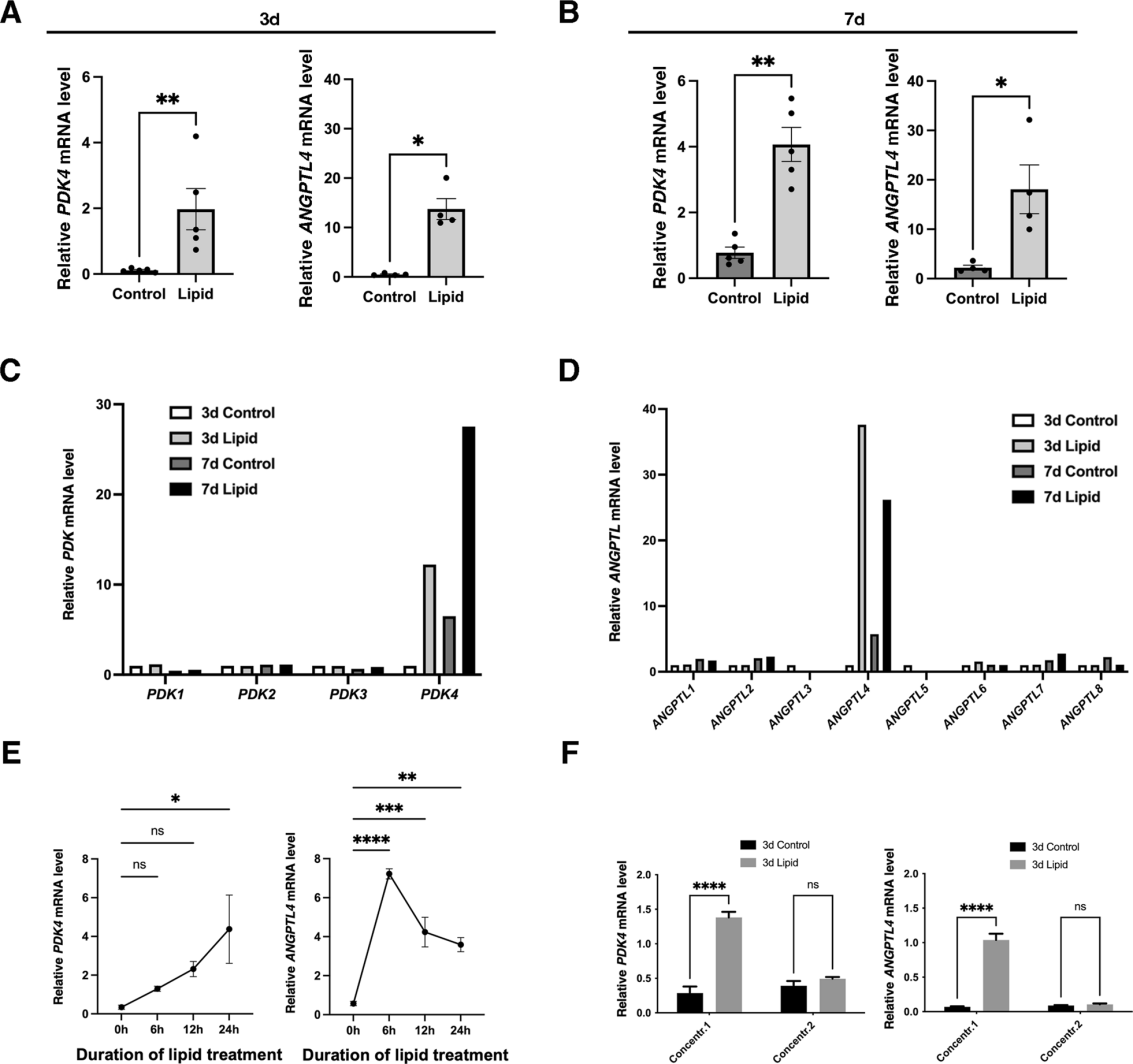
PDK4 and ANGPTL4 are highly induced by lipid treatment in human gastric SMCs. **A** RT-qPCR of *PDK4* and *ANGPTL4* relative mRNA level in human gastric SMC cultures with or without lipid treatment for 3 days. Data were normalized to the house-keeping *HMBS* expression. Values are presented as the mean ± SEM of n = 4–5 samples. Statistically significant differences are indicated as *P < 0.05 and ***P < 0.001 (two-tailed Mann–Whitney test). After 3 days of lipid treatment, *PDK4* and *ANGPTL4* levels were stimulated 13-fold and 29-fold, respectively. **B** RT-qPCR of *PDK4* and *ANGPTL4* relative mRNA level in human gastric SMC cultures with or without lipid treatment for 7 days. Data were normalized to the house-keeping *HMBS* expression. Values are the mean ± SEM of n = 4–5 samples. Statistically significant differences are indicated as *P < 0.05 and ***P < 0.001 (two-tailed Mann–Whitney test). After 7 days of lipid treatment, *PDK4* and *ANGPTL4* levels were stimulated fivefold and eightfold, respectively. **C** Relative mRNA expression of *PDK* members determinates by RNA sequencing from human gastric SMC cultures with or without lipid treatment for 3 and 7 days. Average of n = 3. **D** Relative mRNA expression of *ANGPTL* members determinates by RNA sequencing from human gastric SMC cultures with or without lipid treatment for 3 and 7 days. Average of n = 3. **E** Time-course stimulation of *PDK4* and *ANGPTL4* mRNAs under lipid treatment. RT-qPCR of *PDK4* (left panel), and *ANGPTL4* (right panel) relative mRNA expression in human gastric SMC cultures before treatment (0 h) and after 6, 12 and 24 h with lipid treatment. Values are the mean ± SEM of n = 4 samples. ****P < 0.001; ***P < 0.001; **P < 0.01; *P < 0.05; ns > 0.05 (Ordinary one-way Anova test). PDK4 stimulation is associated with linear increase, where ANGPTL4 harbors a strong induction before a sustained statistic stimulation. **F** RT-qPCR of *PDK4* (left panel), and *ANGPTL4* (right panel) relative mRNA expression in human gastric SMC cultures treated for 3 days with lipid treatment at normal concentration (Concentr. 1), and at dilution 1/4 (Concentr. 2). Data were normalized to house-keeping *HMBS* expression. Values are the mean ± SEM of n = 4 samples. ****P < 0.001; *P < 0.05; ns > 0.05 (Kruskal–Wallis test followed by Dunn’s multiple comparisons test)

To gain a deeper understanding of how lipid treatment mediates these effects, we conducted RNA sequencing analysis on human gastric SMCs after 3 and 7 days of lipid treatment. Using a significance cutoff of p < 0.01, we identified 139 up-regulated and 88 down-regulated genes compared to unstimulated SMCs at both time points without any notable change in pro-inflammatory genes (Fig. 4A; Additional file 2: Figs. 2, 3). Notably, *PDK4* and *ANGPTL4* were found to be strongly upregulated (Fig. 4B-D; Additional file 2: Fig. 2). The stimulation of *PDK4* (Fig. 5A) and *ANGPTL4* (Fig. 5B) transcript level at 3 and 7 days of lipid treatment was confirmed by RT-qPCR. Pyruvate dehydrogenase kinase family proteins (PDKs 1–4) inhibit glycolysis-dependent oxidative phosphorylation (OXPHOS) by inactivating pyruvate dehydrogenase complex, while angiopoietin-like proteins (ANGPTLs 1–8) are involved in various physiological and pathological functions related to tissue repair and homeostasis [35, 36]. Among the PDK and ANGPTL family members, we found only *PDK4* and *ANGPTL4* upregulated in response to lipid treatment (Fig. 5C, D). Moreover, we found that *PDK4* and *ANGPTL4* mRNAs are rapidly induced by lipids treatment in a dose dependent-manner (Figs. 5E, F).

**Fig. 6:**
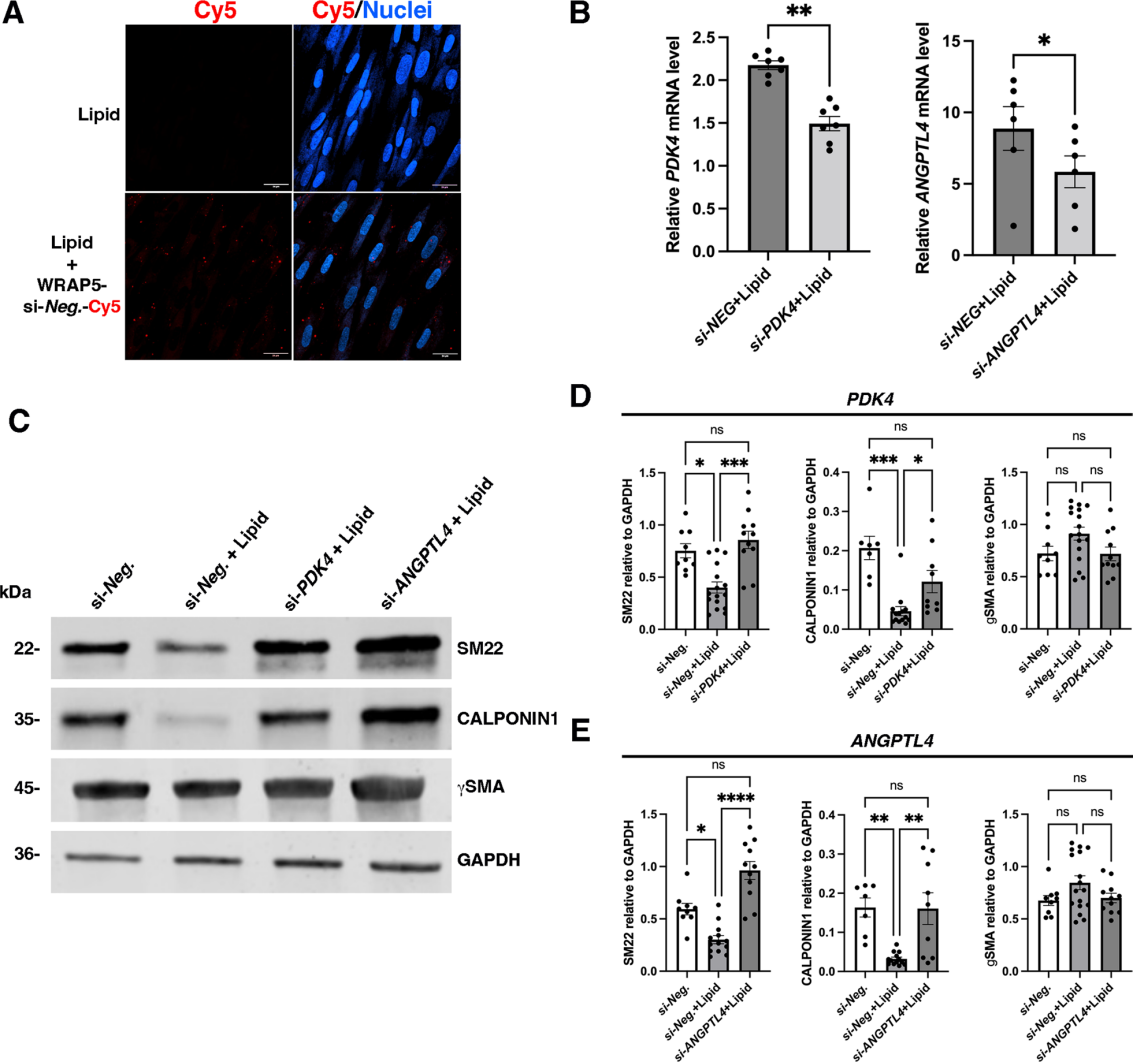
Function of the molecular mechanism induced by lipid treatment in human gastric SMCs. **A** 14-day human gastric SMC cultures were first incubated with WRAP-complexed Scrambled siRNA labeled with Cy5 (WRAP5 + si-Neg.-Cy5) during 2 h then cultures were treated with lipids. Following Hoechst staining, non-fixed cultures were directly analyzed using confocal microscopy. Scale bars: 24 mm. Many red dots detected with Cy5 staining were found in lipid-treated SMCs. **B** RT-qPCR of *PDK4* and *ANGPTL4* relative mRNA expression in human gastric SMC cultures under 3-day lipid treatment with Control siRNA (si-*NEG*) and specific siRNA directed against *PDK4* (si-*PDK4*) and *ANGPTL4* (si-*ANGPTL4*). Data were normalized to house-keeping *HMBS* expression. Values are the mean ± SEM of n = 7 samples. *P < 0.05 and **P < 0.01 (two-tailed Mann–Whitney test). *PDK4* and *ANGPTL4* stimulation are respectively diminished by 34% and 36% after 3 days of respective siRNAs under lipid treatment. **C** Representative Western blot of human gastric SMC extracts treated for 3 days with si-*Neg.* alone (control), si-*Neg.* + lipids, si-*PDK4* + lipids and si-*ANGPTL4* + lipids than probed with antibodies directed against specific smooth muscle proteins (SM22, CALPONIN1, and gSMA) and against GAPDH as loading control. **D** Quantification of Western blot assays comparing extracts treated for 3 days with si-*Neg.* alone (control), si-*Neg.* + lipids, and si-*PDK4* + lipids. Data are presented as the mean ± SEM (nonparametric Kruskall-Wallis test with Dunn’s multiple comparison test: *P < 0.05, ***P < 0.001, ns > 0.05). The expression of SM22 and CALPONIN1 decreased in lipid-treated SMCs compared to the control condition; however, this decrease was abolished in the presence of si-*PDK4*. **E** Quantification of Western blot assays comparing extracts treated for 3 days with si-*Neg.* alone (control), si-*Neg.* + lipids, and si-*ANGPTL4* + lipids. Data are presented as the mean ± SEM (nonparametric Kruskall-Wallis test with Dunn’s multiple comparison test: *P < 0.05, **P < 0.01, ***P < 0.001, ns > 0.05). The expression of SM22 and CALPONIN1 decreased in lipid-treated SMCs compared to the control condition; however, this decrease was abolished in the presence of si-*ANGPTL4*

We investigated the role of *PDK4* and/or *ANGPTL4* upregulation in lipid-induced dedifferentiation of human gastric SMCs. We observed that highly confluent SMCs are difficult to transfect with standard transfection reagents. Moreover, to avoid lipid-based transfection reagents, we employed a peptide-based method for siRNA delivery, using the 16-mer tryptophan- and arginine-rich amphipathic peptide (WRAP) [29]. These peptides form 100 nm nanoparticles with *PDK4* and *ANGPTL4* siRNA molecules (Additional file 2: Fig. 4A, B). We found that Cy5-labeled WRAP5-siRNA nanoparticles were efficiently internalized by nearly all human gastric SMCs within 2 h (Fig. 6A) and did not exhibit cytotoxicity on human gastric SMCs (Additional file 2: Fig. 4C). Targeting *PDK4* or *ANGPTL4* with siRNAs effectively reduced the upregulation induced by lipid treatment (Fig. 6B), maintaining SM22 and CALPONIN1 expression levels similar to those of unstimulated SMCs (Figs. 6C-E).

To further characterize the impact of lipids on SMC dedifferentiation, we extended the duration of lipid exposure to 14 days. Lipid treatment led to a consistent reduction in CALPONIN1 and SM22 expression at both 7 and 14 days, indicating persistent and stable dedifferentiation over time. (Additional file 2: Fig. 5A, B). While *ANGPTL4* induction remained comparable between time points, *PDK4* showed a marked increase over time, suggesting a differential temporal regulation of these two genes by lipids (Additional file 2: Fig. 5C). We next assessed the capacity for phenotypic recovery following lipid removal. After 7 days of lipid treatment, human gastric SMCs were cultured for an additional 7 days in standard medium. *PDK4* and *ANGPTL4* transcript levels returned to baseline upon lipid withdrawal, supporting a reversible induction response to lipid exposure (Additional file 2: Fig. 6A). Under these conditions, SM22 expression also returned to baseline, whereas CALPONIN1 expression remained repressed, indicating a partial restoration of the differentiated phenotype (Additional file 2: Fig. 6B, C). As previously observed, γSMA expression remained unchanged across conditions.

Overall, our findings underscore the crucial role of PDK4 and ANGPTL4 as key regulators in driving the dedifferentiation of human gastric SMCs in the presence of lipids.

### PPARD regulates the induction of PDK4 and ANGPTL4 in lipid-treated gastric SMCs

**Fig. 7:**
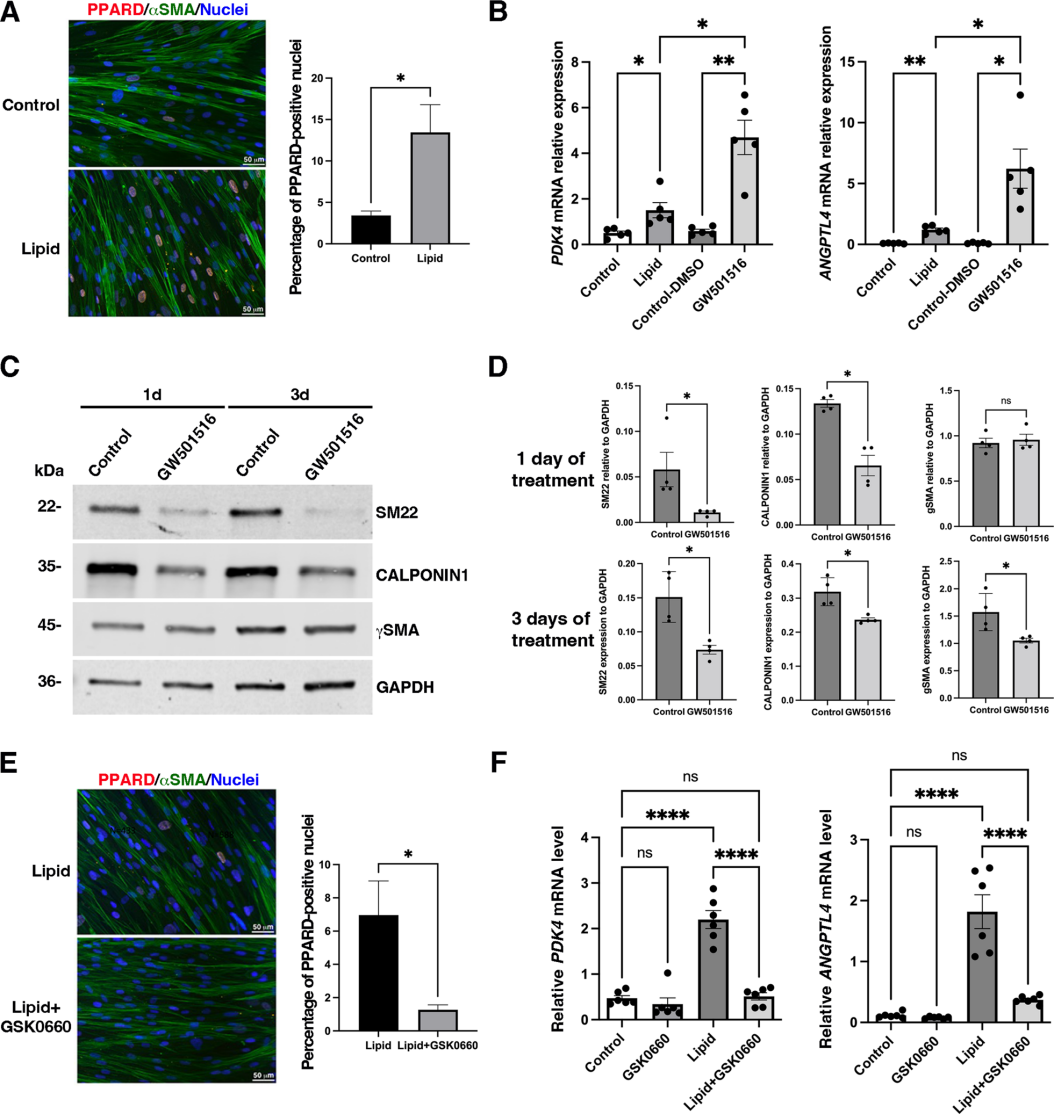
Identification of PPARD activation in the regulation of the expression of *PDK4* and *ANGTPL4* mRNA expression under lipid treatment. **A** Immunofluorescence analysis of human gastric SMC cultures treated with lipid for 2 days compared to untreated controls, stained for PPARD and aSMA. Nuclei were visualized using Hoechst staining. Scale bars: 50 mM. The right panel shows a fourfold increase in the percentage of nuclear PPARD-positive SMCs following lipid treatment. **B** RT-qPCR of *PDK4* (left panel) and *ANGPTL4* (right panel) relative mRNA expression in human gastric SMC cultures treated for 3 days with or without lipid treatment, and with GW501516 (PPARD agonist, 1 mM) or DMSO as Control. Data were normalized to house-keeping *HMBS* expression. Values are the mean ± SEM of n = 5 samples. **P < 0.01; *P < 0.05; ns > 0.05 (One way ANOVA, multiple comparison test). *PDK4* and *ANGPTL4* levels were respectively threefold and fivefold more stimulated in GW501516 condition compared to lipid conditions. **C** Representative Western blot of human gastric SMC extracts from 14 days of culture with or without GW501516 treatment for additive 1 day or 3 days probed with antibodies directed against SM22, CALPONIN1, and gSMA and GAPDH as loading control. **D** Quantification of Western blot assays (n = 6) comparing extracts from GW501516-treated SMCs to extracts from control SMCs. Data are presented as the mean ± SEM and two-tailed Mann–Whitney test was applied (**P < 0.01, *P < 0.05, ns > 0.05). Significant reductions in SM22 and CALPONIN1 protein levels were observed after 1 and 3 days of treatment, with γSMA significantly lower only after 3 days. **E** Immunofluorescence analysis of human gastric SMC cultures treated for 1 day with lipids alone or in combination with GSK0660 (5 mM), stained for PPARD and aSMA. Nuclei were visualized using Hoechst staining. Scale bars: 50 mM. The right panel shows that lipid + GSK0660 treatment reduced the percentage of nuclear PPARD-positive SMCs by sixfold. **F** RT-qPCR of *PDK4* (left panel) and *ANGPTL4* (right panel) relative mRNA levels in human gastric SMC cultures treated for 3 days with GSK0660 (PPARD antagonist, 5 mM), with lipid, and with lipid + GSK0660 compared to untreated SMC (Control). Data were normalized to the house-keeping *HMBS* expression. Values are the mean ± SEM of n = 6 samples. ****P < 0.0001, ns > 0.05 (One way ANOVA, multiple comparison test). *PDK4* and *ANGPTL4* stimulation induced by lipid treatment were significantly reduced when combined with GSK0660, approaching control level

To elucidate the mechanism by which lipid treatment induces *PDK4* and *ANGPTL4* expression, we evaluated whether this effect arises from mRNA stabilization or transcriptional activation. Given our previous findings that RNA-binding proteins can stabilize specific mRNAs to promote SMC dedifferentiation [37], we first investigated potential changes in mRNA stability. Actinomycin D chase experiments revealed that *PDK4* and *ANGPTL4* transcripts were similarly degraded over time in both control and lipid-treated SMCs. No significant differences in mRNA half-life were observed, indicating that lipid-induced upregulation does not result from increased transcript stability, but rather supports a transcriptional mechanism of regulation (Additional file 2: Fig. 7). Peroxisome proliferator-activated receptors (PPARα, PPARγ, and PPARD) are transcription factors that can be activated by fatty acids [38]. We found that PPARD is the most abundantly expressed PPAR member in human gastric SMCs (Additional file 2: Fig. 8A), and lipid treatment induces its nuclear localization (Fig. 7A). PPARs regulate gene expression by forming heterodimers with RXRs and binding to PPAR response elements (PPREs) in the promoter regions of target genes. Notably, PPARD was reported to bind PPREs in the promoters of *ANGPTL4* and *PDK4* in cancer cells, leading to their upregulation [39]. Bioinformatic analysis that we conducted identified conserved PPREs in the promoters of both *PDK4* and *ANGPTL4* (Additional file 2: Fig. 8B).

**Fig. 8:**
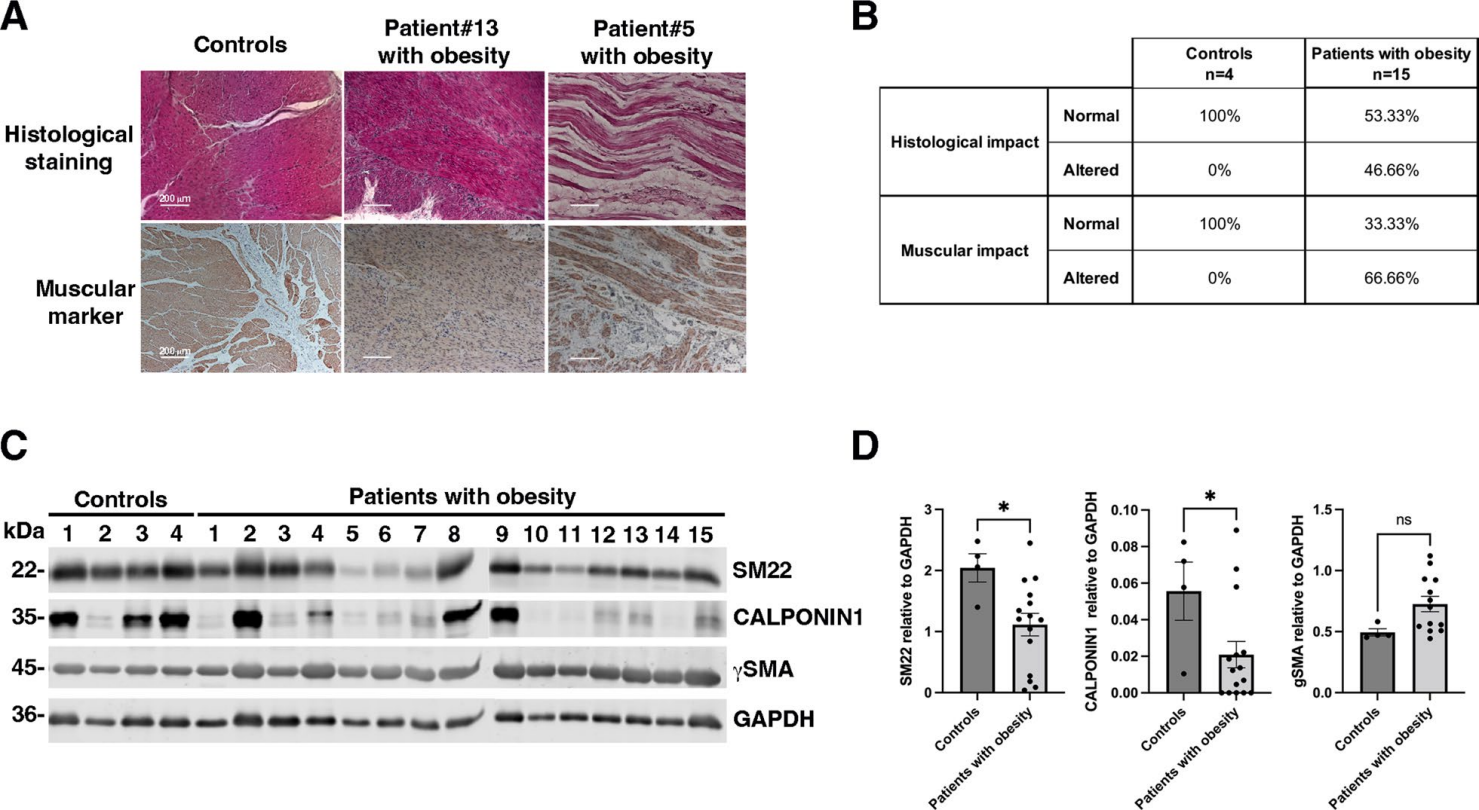
Evaluation of the differentiation status of human gastric smooth muscle in patients with obesity. **A**) Representative Hematoxylin and Eosin staining (upper panels), immunohistochemical staining of smooth muscle marker CALPONIN1 (lower panels) of stomach sections from adult patients with obesity (n = 15), and from lean adults with gastric epithelial cancer (controls; n = 4). 10 of 15 patients with obesity exhibited alteration in the expression and organization of gastric smooth muscle. **B** Summary of the histological evaluation of stomach sections from adult patients with obesity (n = 15) and adult controls (n = 4) using Hematoxylin and Eosin staining, along with immunohistochemical staining for the smooth muscle marker CALPONIN1. After staining, gastric smooth muscle was evaluated and scored independently for their organization into normal, and affected regions by three pathologists and consensus was reported. **C** Western-blotting of gastric smooth muscle fiber extracts from adult patients with obesity (n = 15), and from controls (n = 4) probed with antibodies directed against specific smooth muscle proteins (SM22, CALPONIN1, and gSMA) and against GAPDH as loading control. **D** Quantification of Western-blot assays (**C**) comparing extracts from adult patients with obesity (n = 15) to extracts from controls (n = 4). Data are presented as the mean ± SEM (two-tailed Mann–Whitney test; *P < 0.05). Notably, the expression of SM22 and CALPONIN1 were found significantly lower in patients with obesity compared to controls

We then evaluated the consequence of direct activation of PPARD in differentiated SMCs using its selective agonist GW501516. This led to a significantly increase in *PDK4* and *ANGPTL4* mRNA expression, and to a greater extent than lipid treatment (Fig. 7B). This phenotype was associated with a reduction in SM22 and CALPONIN1 levels after just one day of treatment, while γSMA expression remained unchanged (Fig. 7C, D). Interestingly, after three days of GW501516 treatment, not only SM22 and CALPONIN1 levels are impacted, but γSMA expression also decreased (Fig. 7C, D), suggesting a more pronounced induction of SMC dedifferentiation. Additionally, GSK0660, a reversible PPARD antagonist, inhibited the lipid-induced nuclear accumulation of PPARD (Fig. 7E) and decreased the upregulation of *PDK4* and *ANGPTL4* mRNA (Fig. 7F). Treatment with GSK3787, a covalent PPARD antagonist, inhibits the lipid-induced upregulation of both *PDK4* and *ANGPTL4* mRNAs (Additional file 2: Fig. 9A) and conducts to a partial restoration of the differentiated phenotype, with a significant increase in SM22 expression, while CALPONIN1 expression remained repressed, indicating incomplete reactivation of the mature SMC program (Additional file 2: Fig. 9B, C).

**Fig. 9:**
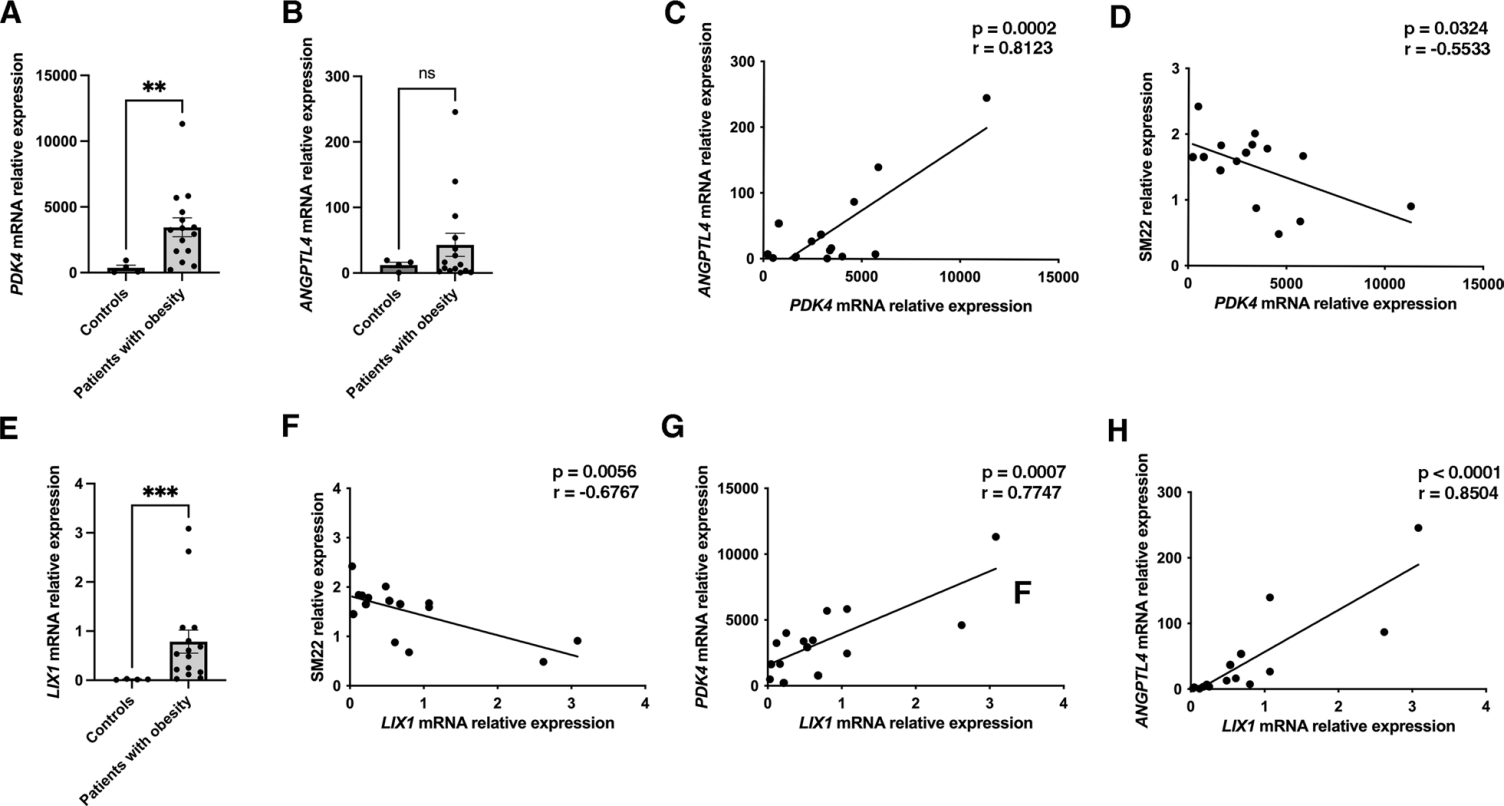
Evaluation of the expression of *PDK4*, *ANGPTL4* and *LIX1* expression and their correlation in patients with obesity. **A** Relative mRNA level of *PDK4* in gastric smooth muscle fiber extracts from adult patients with obesity (n = 15) compared to controls (n = 4) was assessed by RT-qPCR. Data were normalized to the levels of the housekeeping gene *HMBS*. Values are presented as mean ± SEM, and statistical analysis was performed using a two-tailed Mann–Whitney test (**P < 0.01). *PDK4* level was found to be 13-fold upregulated in patients with obesity. **B** Relative mRNA expression of *ANGPTL4* in gastric smooth muscle fiber extracts from adult patients with obesity (n = 15) and controls (n = 4) was evaluated by RT-qPCR. Data were normalized to *HMBS* level, presented as mean ± SEM, and analyzed using a two-tailed Mann–Whitney test (ns > 0.05). *ANGPTL4* level showed a 3.5-fold increase in patients with obesity. **C**, **D** The correlation between *PDK4* and *ANGPTL4* levels (**C**), as well as between *PDK4* level and SM22 expression **D**, was analyzed using Pearson’s correlation test. P and R values are indicated on each graph. Positive correlation was observed between *PDK4* and *ANGPTL4*, while a negative correlation was identified between *PDK4* and SM22. **E** Relative mRNA level of *LIX1* in gastric smooth muscle fiber extracts from adult patients with obesity (n = 15) and controls (n = 4) was assessed by RT-qPCR. Data were normalized to *HMBS* level, presented as mean ± SEM, and analyzed using a two-tailed Mann–Whitney test (***P < 0.001). *LIX1* level was found to be 22-fold upregulated in patients with obesity. (F–H) The correlation between *LIX1* and SM22 expression (**F**), or *PDK4* (**G**) and *ANGPTL4* (**H**) levels were calculated by Pearson’s correlation test. P and R values are indicated on each graph. A negative correlation was observed between *LIX1* and SM22 expression, while positive correlations were observed between *LIX1* and both *PDK4* and *ANGPTL4*

These findings highlight the significant role of PPARD activity in regulating *PDK4* and *ANGPTL4* mRNA, which in turn drive the dedifferentiation of human gastric SMCs in response to lipid stimuli.

### PDK4 and ANGPTL4 expression correlates to gastric smooth muscle dedifferentiation and the acquisition of immature features in patients with obesity

To investigate potential changes in gastric musculature in patients with obesity, we first analyzed with histopathology and immunohistochemistry approaches 15 samples from patients who underwent laparoscopic sleeve gastrectomy for obesity, as well as 4 samples from lean patients associated with gastric or esophageal carcinoma (controls) (Additional file 1: Tables S1, S2). We found that 66% of patients with obesity showed changes in the organization of the smooth muscle layers, while no alterations were found in the control subjects (Fig. 8A, B). Next, we evaluated the differentiation status of gastric SMCs using CALPONIN1 and SM22 marker antibodies. Western blot analysis of extracts from dissected gastric smooth muscle fibers confirms a significant decrease in the expression levels of differentiated markers, such as SM22 and CALPONIN1 in patients with obesity compared to controls (Fig. 8C, D). As previously observed in mice and in SMC culture, the expression level of gSMA, a marker defining SMC determination and identity, remained unchanged indicating a specific deregulation of the differentiation status of the SMCs (Fig. 8C, D).

**Fig. 10:**
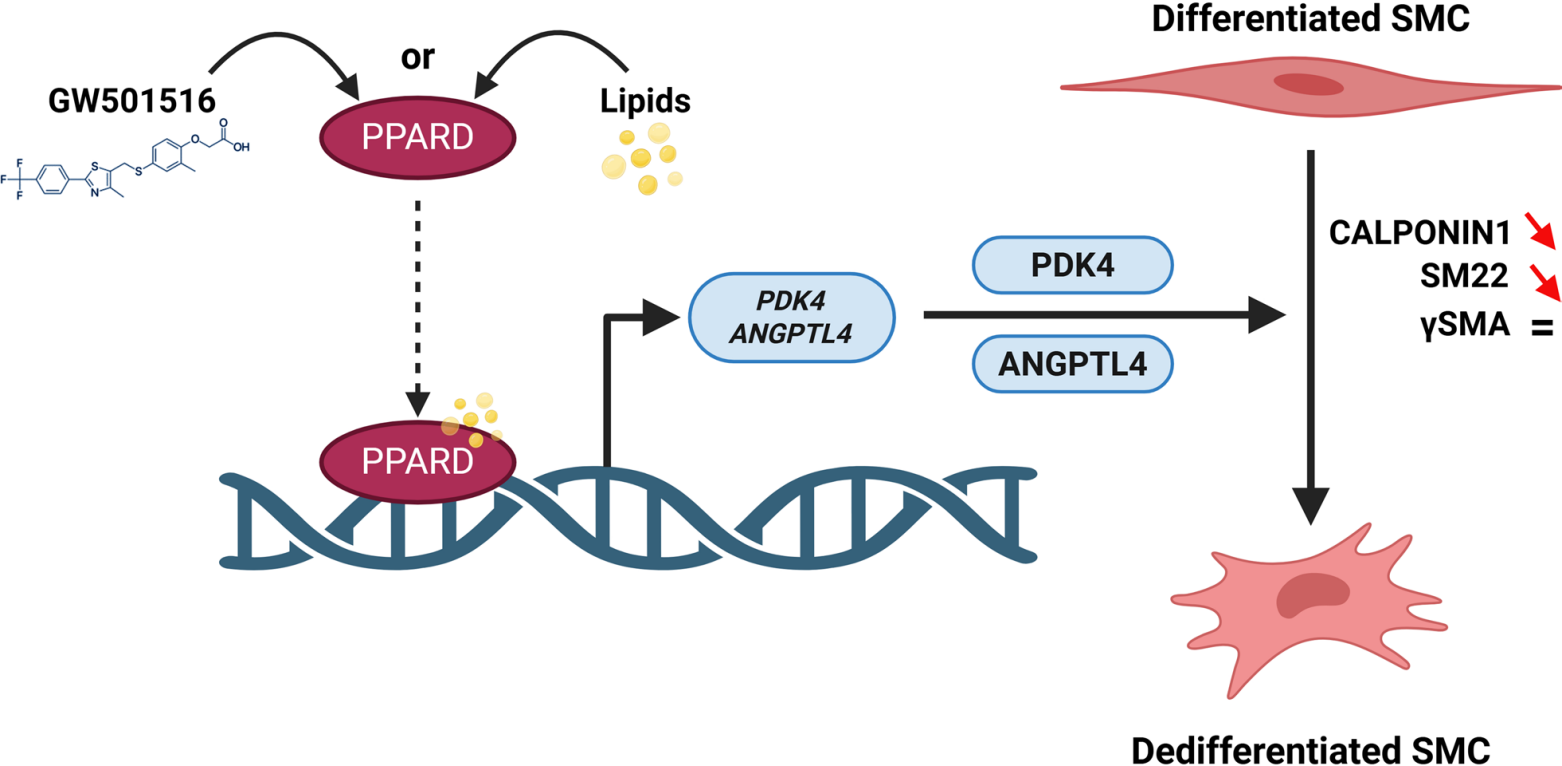
Schematic model of the pathway leading to alterations in gastric smooth muscle in patients with obesity. Upon lipid exposure or specific agonist (GW501516), the ligand-activated transcription factor PPARD becomes active. These regulations correlate with the nuclear translocation shuttling of the PPARD protein, supporting a potential transcriptional control at the peroxisome proliferator response elements (PPREs) of *PDK4* and *ANGPTL4* promoter, which still needs to be validated in our model. Upregulation of PDK4 and ANGPTL4 is crucial for initiating the dedifferentiation process of gastric SMCs observed in patients with obesity

We subsequently quantified the transcript levels of *PDK4* and *ANGPTL4*. Our analysis revealed that *PDK4* mRNA is significantly upregulated in patients with obesity compared to controls (Fig. 9A). Although *ANGPTL4* mRNA levels showed an increase, this change did not reach statistical significance (Fig. 9B). Furthermore, a significant positive correlation was observed between *ANGPTL4* and *PDK4* mRNA levels in patients with obesity, suggesting a link between their expression (Fig. 9C). Conversely, an inverse correlation was found between *PDK4* mRNA and SM22 protein levels (Fig. 9D). Considering that alterations in smooth muscle differentiation are often associated with mesenchymal immaturity, we investigated the expression of LIX1, a specific marker and regulator of stomach mesenchymal progenitors[21, 22]. Our analysis revealed a significant upregulation of *LIX1* mRNA in patients with obesity compared to controls (Fig. 9E). Additionally, in patients with obesity, *LIX1* mRNA levels demonstrated an inverse correlation with SM22 protein expression (Fig. 9F). Notably, LIX1 levels showed significant positive correlations with both *PDK4* and *ANGPTL4* mRNA expressions (Figs. 9G, H). These findings suggest a link between the expressions of *PDK4* and *ANGPTL4* and the immaturity of gastric smooth muscle in patients with obesity (Fig. 10).

## Discussion

The stomach plays a central role in regulating food intake through its capacity to store, mix, propel, and empty its contents, all of which require highly coordinated motor function [16]. In individuals with obesity, accelerated gastric emptying has been associated with significant weight gain [5, 12, 13]. However, the mechanisms underlying gastric emptying dysfunction in these patients remain unclear. Gastric emptying, the process of moving stomach contents into the small intestine, is driven by peristaltic contractions—rhythmic waves of SMC activity that propel chyme toward the pylorus. Effective peristalsis requires fully differentiated SMCs for proper contractile function. We hypothesize that impaired differentiation of SMCs may contribute to the gastric emptying dysfunction observed in individuals with obesity.

In this study, we analyzed human stomach samples from patients with obesity and mouse model of HFD-induced obesity to assess the differentiation status of SMCs. Our analyses of both models revealed reduced levels of the differentiation markers SM22 and CALPONIN1 in their SMCs under obese conditions compared to controls. In contrast, the expression of γSMA, a marker of SMC determination and identity, remained unchanged, indicating a specific dysregulation of the differentiation status of these cells. Additionally, we observed elevated expression of LIX1 in the gastric smooth muscle of patients with obesity, a gene typically restricted to mesenchymal progenitors during fetal development [21, 22]. One limitation of our study is the use of a single mouse strain (C57BL/6) and the exclusive analysis of male animals, which does not account for potential sex-specific differences in gastric smooth muscle response to HFD treatment, differences that may involve estrogen signaling pathways, including the sex-dependent expression of the G protein–coupled estrogen receptor (GPER) recently linked to gastrointestinal dysmotility in female diabetic mice [40–42]. However, our combined findings suggest a dedifferentiation process [18, 19], akin to mechanisms observed in pediatric and adult functional dysmotility syndromes, including primary visceral myopathy, inflammatory bowel disease, and achalasia [18–20]. Therefore, although gastric SMCs retain their identity, they may lose their ability to contract under conditions of obesity.

The stomach is a complex organ composed of various cell types, including epithelial cells, enteric neurons, glial cells, and mesenchymal-derived cells such as SMCs and ICCs [6, 14]. HFD-induced obesity has been shown to prevent age-associated loss in specific populations of enteric neurons, contributing to accelerated gastric emptying [32]. In the intestine, HFD-induced obesity increases the numbers and function of Lgr5 + intestinal epithelial stem cells in mammals [31]. Recent studies have investigated the immediate responses of the intestinal epithelium to HFD, revealing metabolic alterations that occur within days of exposure, leading to rapid epithelial adaptation [43]. Given the well-established requirement of epithelial-mesenchymal interactions in both the development and homeostasis of the GI tract [6, 19, 44], the dedifferentiation of gastric SMCs may arise from changes in the gastric epithelium. An alternative explanation could be a direct effect of lipids on gastric SMCs following their distribution through the GI vasculature. To distinguish between these two hypotheses, we first developed an in vitro model of differentiated human gastric SMCs that we treated with a lipid mixture enriched in oleic and palmitic acids. This lipid treatment had previously been used to demonstrate the impact of lipids on the intestinal epithelium [31]. We showed that this treatment caused a phenotypic change in SMCs, marked by a specific reduction in differentiation markers, indicating a direct effect of lipids on SMCs. The phenotypic changes in SMCs observed in vitro—specifically, the loss of the differentiation markers CALPONIN1 and SM22, along with the maintenance of γSMA expression—are similar to those seen in patients with obesity and in HFD-induced mice. This highlights the contribution of a direct impact of lipids on SMCs in patients with obesity.

Using the previously mentioned myogenic model, we investigated the signaling mechanisms leading to SMC dedifferentiation in response to lipids. Our findings emphasized the pivotal role of a signaling cascade activated by lipid stimulation, in which PPARD regulates the mRNA levels of *PDK4* and *ANGPTL4*. PPARs are transcription factors that modulate gene expression by forming heterodimers with RXRs and binding to PPREs in the promoters of target genes, and they can be activated by various ligands, especially fatty acids [39]. Previous research has demonstrated that PPARD is expressed during the development of the vertebrate GI mesenchyme [45]. Our data revealed that PPARD is the most highly expressed member of the PPAR family in human gastric SMCs, and its nuclear accumulation is rapidly induced upon lipid exposure. By employing specific PPARD agonist, we observed that direct PPARD activation resulted in a rapid decrease in SM22 and CALPONIN1 expression, mirroring the phenotypic changes seen after lipid treatment. We also established that PPARD agonist treatment is sufficient to upregulate *PDK4* and *ANGPTL4* mRNA. Moreover, we demonstrated using reversible and covalent PPARD antagonists that PPARD activation following lipids treatment is required for *PDK4* and *ANGPTL4* mRNA induction. These regulations correlate with the nuclear translocation shuttling of the PPARD protein, supporting a potential transcriptional control at the promoter level, which still needs to be validated in our system (Fig. 10). Our recovery experiments revealed that *PDK4* and *ANGPTL4* mRNA levels return to baseline following lipid removal, indicating a reversible activation of the PPARD signaling pathway. However, SMC redifferentiation remained incomplete, with normalization of SM22 expression (early SMC differentiation marker), but persistent suppression of CALPONIN1, a marker of late SMC differentiation. This partial recovery phenotype is also observed, when lipid treatment was associated with PPARD antagonist GSK3787. One possibility is that reactivation of the full differentiation program, particularly for late markers, may require extended recovery periods or the addition of pro-differentiation cues. In this context, stimulation of contractile activity, which has been shown to be essential for both SMC differentiation initiation [15, 46] and the maintenance of their status in the digestive tract [47], could represent a relevant signal to restore the mature SMC phenotype. Moreover, our findings suggest that although PPARD signaling is necessary for the dedifferentiation process, its inhibition alone may be insufficient to fully prevent or reverse the lipid-induced phenotype, pointing to the involvement of additional signaling pathways, such as lipid-driven post-translational modifications, in sustaining SMC reprogramming. To further investigate the causal role of PPARD in gastric smooth muscle dysfunction during obesity, future studies will employ a conditional knockout strategy using PPARD-flox/flox mice crossed with smooth muscle–specific Cre lines (e.g., Sm22-Cre, Myh11-CreERT2), and potentially Bapx1-Cre for gastric specificity [48], to selectively ablate PPARD in gastric smooth muscle cells under HFD conditions. These findings are consistent with studies in other cell types, such as skeletal muscle and adipocytes, indicating that PPARD may regulate *PDK4* and/or *ANGPTL4* [49, 50].

PDK4, a crucial regulator of the pyruvate dehydrogenase complex, significantly contributes to obesity-related insulin resistance and metabolic dysfunction [51]. Its elevated activity in obesity enhances the formation of mitochondria-associated endoplasmic reticulum membranes while suppressing insulin signaling [51]. PDK4 is expressed in skeletal muscle and the heart, with notably higher levels in the skeletal muscle of insulin-resistant individuals [52]. In patients with obesity, reduced promoter methylation of *PDK4* is observed in skeletal muscle; however, this methylation returns to levels similar to those observed in non-obese individuals following weight loss from Roux-en-Y gastric bypass, indicating an inverse relationship with *PDK4* mRNA expression [53]. ANGPTL4, which is essential for lipid metabolism, regulates lipoprotein lipase activity in various tissues [54]. Induced by fasting, ANGPTL4 is predominantly expressed in the liver, adipose tissue, and ischemic tissues [36]. Individuals carrying the pE40K variant of *ANGPTL4* have lower triglyceride levels, higher high-density lipoprotein (HDL) cholesterol [55], and a reduced risk of coronary artery disease [56]. Moreover, these individuals demonstrate lower fasting glucose levels, improved insulin sensitivity, and a decreased likelihood of developing type 2 diabetes [57]. While numerous studies have linked serum levels of ANGPTL4 to body mass index, particularly in the context of type 2 diabetes, the source and functional significance of ANGPTL4 expression remain poorly understood.

PDK4 and ANGPTL4 expression had not been previously evaluated in human gastric smooth muscle. We found that *PDK4* and *ANGPTL4* transcript levels were upregulated in SMCs in response to lipid exposure or when PPARD activation was sustained in SMCs using its selective agonist, GW501516. Targeting *PDK4* or *ANGPTL4* with specific siRNAs reversed the phenotypic changes observed in SMCs following lipid stimulation, confirming their critical roles in lipid-induced SMC dedifferentiation. Importantly, we found a significant increase in *PDK4* expression in gastric smooth muscle fibers from patients with obesity compared to controls, while *ANGPTL4* levels showed a slight but non-significant increase. Furthermore, we reported a significant positive correlation between the digestive mesenchymal progenitor marker *LIX1* and both *PDK4* and *ANGPTL4* expression. Therefore, these findings may have a clinical relevance as the expression of *PDK4* and *ANGPTL4* correlates with gastric smooth muscle dedifferentiation and the development of immature features in patients with obesity. These findings emphasize the critical role of the PDK4/ANGPTL4 pathway in gastric smooth muscle cell dedifferentiation in obesity and suggest a novel mechanism that could contribute to gastric muscle dysfunction, ultimately leading to accelerated gastric emptying [4, 9–12]. We have previously shown that gastric SMC differentiation is tightly regulated by mitochondrial metabolism, particularly via LIX1-dependent mechanisms [22]. Consistent with this, both PDK4 and ANGPTL4 are key regulators of energy and lipid metabolism with established roles in mitochondrial function. PDK4 inhibits the pyruvate dehydrogenase complex, thereby modulating mitochondrial oxidative activity, while ANGPTL4 regulates lipoprotein lipase activity and may indirectly influence mitochondrial lipid handling. Moreover, our results highlight a potential shift from an early ANGPTL4-dependent response to a longer-term PDK4-driven metabolic reprogramming sustaining SMC dedifferentiation. These observations highlight mitochondrial reprogramming as a central mechanism driving gastric SMC dedifferentiation and support the relevance of prioritizing metabolic pathways over inflammatory ones in our current model.

## Conclusions

This study reveals a novel mechanistic link between obesity and gastric smooth muscle dysfunction, implicating the activation of the PPARD/PDK4/ANGPTL4 pathway. The correlation of *PDK4* and *ANGPTL4* expression with markers of mesenchymal immaturity and smooth muscle dedifferentiation underscores their potential as biomarkers and targets for therapeutic intervention in obesity-related gastric dysmotility. Although targeting PPARD, PDK4, and ANGPTL4 holds therapeutic promise, their clinical application requires the development of smooth muscle–specific delivery strategies—such as peptide-functionalized nanoparticles—to avoid off-target effects. Moreover, further studies are needed to assess whether smooth muscle dedifferentiation and the upregulation of these pathways are also present in other obesity-related gastrointestinal disorders (e.g., GERD, functional dyspepsia), and whether they correlate with impaired gastric motility and SMC dysfunction as observed in obesity. These insights open promising avenues for the development of targeted therapeutic strategies to mitigate the GI disorders associated with obesity.

## Supplementary Information

Additional file 1

Additional file 2

## Data Availability

All data generated or analyzed during this study are included in this published article and its additional files. The data used in the current study are available from the corresponding authors upon reasonable request.

## Abbreviations

ANGPTL: Angiopoietin-related protein
GI: Gastrointestinal
HFD: High-fat diet
LIX1: Limb expression 1
ICC: Interstitial cells of Cajal
PDK: Pyruvate dehydrogenase kinase
PPAR: Peroxisome proliferator-activated receptor
RT-qPCR: Quantitative Reverse Transcription PCR
siRNA: Small interfering RNA
aSMA: Alpha smooth muscle actin
gSMA: Gamma smooth muscle actin
SMCs: Smooth muscle cells
WRAP: Tryptophan- and arginine-rich amphipathic peptide

## References

[1] NCD Risk Factor Collaboration (NCD-RisC). Worldwide trends in underweight and obesity from 1990 to 2022: a pooled analysis of 3663 population-representative studies with 222 million children, adolescents, and adults. Lancet. 2024;403:1027–50. 38432237 10.1016/S0140-6736(23)02750-2 PMC7615769

[2] Afshin A, Reitsma MB, Murray CJL. Health Effects of Overweight and Obesity in 195 Countries. N Engl J Med. 2017;377:1496–7. 29020584 10.1056/NEJMc1710026

[3] Acosta A, Abu Dayyeh BK, Port JD, Camilleri M. Recent advances in clinical practice challenges and opportunities in the management of obesity. Gut. 2014;63:687–95. 24402654 10.1136/gutjnl-2013-306235 PMC4170188

[4] Acosta A, Camilleri M. Gastrointestinal morbidity in obesity. Ann N Y Acad Sci. 2014;1311:42–56. 24602085 10.1111/nyas.12385 PMC4122663

[5] Delgado-Aros S, Locke GR, Camilleri M, Talley NJ, Fett S, Zinsmeister AR, et al. Obesity is associated with increased risk of gastrointestinal symptoms: a population-based study. Am J Gastroenterol. 2004;99:1801–6. 15330922 10.1111/j.1572-0241.2004.30887.x

[6] de Santa BP, van den Brink GR, Roberts DJ. Development and differentiation of the intestinal epithelium. Cell Mol Life Sci. 2003;60:1322–32. 12943221 10.1007/s00018-003-2289-3 PMC2435618

[7] Steenackers N, Eksteen G, Wauters L, Augustijns P, Van der Schueren B, Vanuytsel T, et al. Understanding the gastrointestinal tract in obesity: From gut motility patterns to enzyme secretion. Neurogastroenterol Motil. 2024;36: e14758. 38342973 10.1111/nmo.14758

[8] Chen J, Chen L, Sanseau P, Freudenberg JM, Rajpal DK. Significant obesity-associated gene expression changes occur in the stomach but not intestines in obese mice. Physiol Rep. 2016;4: e12793. 27207783 10.14814/phy2.12793 PMC4886165

[9] Wright RA, Krinsky S, Fleeman C, Trujillo J, Teague E. Gastric emptying and obesity. Gastroenterology. 1983;84:747–51. 6825986

[10] Christian PE, Datz FL, Moore JG. Gastric emptying studies in the morbidly obese before and after gastroplasty. J Nucl Med. 1986;27:1686–90. 3772503

[11] Acosta A, Camilleri M, Burton D, O’Neill J, Eckert D, Carlson P, et al. Exenatide in obesity with accelerated gastric emptying: a randomized, pharmacodynamics study. Physiol Rep. 2015;3: e12610. 26542264 10.14814/phy2.12610 PMC4632965

[12] Camilleri M, Malhi H, Acosta A. Gastrointestinal complications of obesity. Gastroenterology. 2017;152:1656–70. 28192107 10.1053/j.gastro.2016.12.052 PMC5609829

[13] Pajot G, Camilleri M, Calderon G, Davis J, Eckert D, Burton D, et al. Association between gastrointestinal phenotypes and weight gain in younger adults: a prospective 4-year cohort study. Int J Obes (Lond). 2020;44:2472–8. 32415254 10.1038/s41366-020-0593-8 PMC7666652

[14] Ward SM, Sanders KM. Physiology and pathophysiology of the interstitial cell of Cajal: from bench to bedside. I. Functional development and plasticity of interstitial cells of Cajal networks. Am J Physiol Gastrointest Liver Physiol. 2001;281:602–11. 10.1152/ajpgi.2001.281.3.G602 11518672

[15] Chevalier NR. The first digestive movements in the embryo are mediated by mechanosensitive smooth muscle calcium waves. Philos Trans R Soc Lond B Biol Sci. 2018;373:20170322. 30249773 10.1098/rstb.2017.0322 PMC6158196

[16] Di Natale MR, Athavale ON, Wang X, Du P, Cheng LK, Liu Z, et al. Functional and anatomical gastric regions and their relations to motility control. Neurogastroenterol Motil. 2023;35: e14560. 36912719 10.1111/nmo.14560

[17] Hayashi Y, Toyomasu Y, Saravanaperumal SA, Bardsley MR, Smestad JA, Lorincz A, et al. Hyperglycemia increases interstitial cells of cajal via MAPK1 and MAPK3 signaling to ETV1 and KIT. Lead Rapid Gastric Empt Gastroenterol. 2017;153:521-535.e20. 10.1053/j.gastro.2017.04.020 PMC5526732 28438610

[18] Scirocco A, Matarrese P, Carabotti M, Ascione B, Malorni W, Severi C. Cellular and molecular mechanisms of phenotypic switch in gastrointestinal smooth muscle. J Cell Physiol. 2016;231:295–302. 26206426 10.1002/jcp.25105

[19] Le Guen L, Marchal S, Faure S, de Santa BP. Mesenchymal-epithelial interactions during digestive tract development and epithelial stem cell regeneration. Cell Mol Life Sci. 2015;72:3883–96. 26126787 10.1007/s00018-015-1975-2 PMC5395663

[20] Martire D, Garnier S, Sagnol S, Bourret A, Marchal S, Chauvet N, et al. Phenotypic switch of smooth muscle cells in paediatric chronic intestinal pseudo-obstruction syndrome. J Cell Mol Med. 2021;25:4028–39. 33656779 10.1111/jcmm.16367 PMC8051695

[21] McKey J, Martire D, de Santa BP, Faure S. LIX1 regulates YAP1 activity and controls the proliferation and differentiation of stomach mesenchymal progenitors. BMC Biol. 2016;14:34. 27125505 10.1186/s12915-016-0257-2 PMC4848777

[22] Guérin A, Angebault C, Kinet S, Cazevieille C, Rojo M, Fauconnier J, et al. LIX1-mediated changes in mitochondrial metabolism control the fate of digestive mesenchyme-derived cells. Redox Biol. 2022;56: 102431. 35988446 10.1016/j.redox.2022.102431 PMC9420520

[23] Wang Z, Wang D-Z, Pipes GCT, Olson EN. Myocardin is a master regulator of smooth muscle gene expression. Proc Natl Acad Sci USA. 2003;100:7129–34. 12756293 10.1073/pnas.1232341100 PMC165841

[24] Struijs M-C, Diamond IR, de Silva N, Wales PW. Establishing norms for intestinal length in children. J Pediatr Surg. 2009;44:933–8. 19433173 10.1016/j.jpedsurg.2009.01.031

[25] Nair DG, Miller KG, Lourenssen SR, Blennerhassett MG. Inflammatory cytokines promote growth of intestinal smooth muscle cells by induced expression of PDGF-Rβ. J Cell Mol Med. 2014;18:444–54. 24417820 10.1111/jcmm.12193 PMC3955151

[26] Rouleau C, Matécki S, Kalfa N, Costes V, de Santa BP. Activation of MAP kinase (ERK1/2) in human neonatal colonic enteric nervous system. Neurogastroenterol Motil. 2009;21:207–14. 18798794 10.1111/j.1365-2982.2008.01187.x PMC2913054

[27] Meziat C, Boulghobra D, Strock E, Battault S, Bornard I, Walther G, et al. Exercise training restores eNOS activation in the perivascular adipose tissue of obese rats: Impact on vascular function. Nitric Oxide. 2019;86:63–7. 30836135 10.1016/j.niox.2019.02.009

[28] Frankel D, Davies M, Bhushan B, Kulaberoglu Y, Urriola-Munoz P, Bertrand-Michel J, et al. Cholesterol-rich naked mole-rat brain lipid membranes are susceptible to amyloid beta-induced damage in vitro. Aging (Albany NY). 2020;12:22266–90. 33147569 10.18632/aging.202138 PMC7695401

[29] Konate K, Josse E, Tasic M, Redjatti K, Aldrian G, Deshayes S, et al. WRAP-based nanoparticles for siRNA delivery: a SAR study and a comparison with lipid-based transfection reagents. J Nanobiotechnology. 2021;19:236. 34380479 10.1186/s12951-021-00972-8 PMC8359084

[30] Qiu B, Simon MC. BODIPY 493/503 staining of neutral lipid droplets for microscopy and quantification by flow cytometry. Bio Protoc. 2016;6: e1912. 28573161 10.21769/BioProtoc.1912 PMC5448404

[31] Beyaz S, Mana MD, Roper J, Kedrin D, Saadatpour A, Hong S-J, et al. High-fat diet enhances stemness and tumorigenicity of intestinal progenitors. Nature. 2016;531:53–8. 26935695 10.1038/nature17173 PMC4846772

[32] Baudry C, Reichardt F, Marchix J, Bado A, Schemann M, Varannes SB, et al. Diet-induced obesity has neuroprotective effects in murine gastric enteric nervous system: involvement of leptin and glial cell line-derived neurotrophic factor. J Physiol. 2012;590:533–44. 22124147 10.1113/jphysiol.2011.219717 PMC3379699

[33] Faure S, McKey J, Sagnol S, de Santa BP. Enteric neural crest cells regulate vertebrate stomach patterning and differentiation. Development. 2015;142:331–42. 25519241 10.1242/dev.118422

[34] Vaes RDW, van den Berk L, Boonen B, van Dijk DPJ, Olde Damink SWM, Rensen SS. A novel human cell culture model to study visceral smooth muscle phenotypic modulation in health and disease. Am J Physiol Cell Physiol. 2018;315:C598-607. 30044660 10.1152/ajpcell.00167.2017

[35] Atas E, Oberhuber M, Kenner L. The implications of PDK1-4 on tumor energy metabolism. Aggress Therapy Resist Front Oncol. 2020;10: 583217. 33384955 10.3389/fonc.2020.583217 PMC7771695

[36] Sylvers-Davie KL, Davies BSJ. Regulation of lipoprotein metabolism by ANGPTL3, ANGPTL4, and ANGPTL8. Am J Physiol Endocrinol Metab. 2021;321:E493-508. 34338039 10.1152/ajpendo.00195.2021 PMC8560382

[37] Sagnol S, Yang Y, Bessin Y, Allemand F, Hapkova I, Notarnicola C, et al. Homodimerization of RBPMS2 through a new RRM-interaction motif is necessary to control smooth muscle plasticity. Nucleic Acids Res. 2014;42:10173–84. 25064856 10.1093/nar/gku692 PMC4150794

[38] Berger J, Moller DE. The mechanisms of action of PPARs. Annu Rev Med. 2002;53:409–35. 11818483 10.1146/annurev.med.53.082901.104018

[39] Legrand N, Bretscher CL, Zielke S, Wilke B, Daude M, Fritz B, et al. PPARβ/δ recruits NCOR and regulates transcription reinitiation of ANGPTL4. Nucleic Acids Res. 2019;47:9573–91. 31428774 10.1093/nar/gkz685 PMC6765110

[40] Org E, Mehrabian M, Parks BW, Shipkova P, Liu X, Drake TA, et al. Sex differences and hormonal effects on gut microbiota composition in mice. Gut Microbes. 2016;7:313–22. 27355107 10.1080/19490976.2016.1203502 PMC4988450

[41] Lefebvre C, Tiffay A, Breemeersch C-E, Dreux V, Bôle-Feysot C, Guérin C, et al. Sex-dependent effects of a high fat diet on metabolic disorders, intestinal barrier function and gut microbiota in mouse. Sci Rep. 2024;14:19835. 39191839 10.1038/s41598-024-70931-4 PMC11349972

[42] Muhammad A, Hixon JC, Pharmacy Yusuf A, Rivas Zarete JI, Johnson I, Miller J, et al. Sex-specific epigenetics drive low GPER expression in gastrointestinal smooth muscles in type 2 diabetic mice. Sci Rep. 2024;14:5633. 38453938 10.1038/s41598-024-54213-7 PMC10920797

[43] Enriquez JR, McCauley HA, Zhang KX, Sanchez JG, Kalin GT, Lang RA, et al. A dietary change to a high-fat diet initiates a rapid adaptation of the intestine. Cell Rep. 2022;41: 111641. 36384107 10.1016/j.celrep.2022.111641 PMC9817065

[44] McLin VA, Henning SJ, Jamrich M. The role of the visceral mesoderm in the development of the gastrointestinal tract. Gastroenterology. 2009;136:2074–91. 19303014 10.1053/j.gastro.2009.03.001

[45] Hojo M, Takada I, Kimura W, Fukuda K, Yasugi S. Expression patterns of the chicken peroxisome proliferator-activated receptors (PPARs) during the development of the digestive organs. Gene Expr Patterns. 2006;6:171–9. 16325478 10.1016/j.modgep.2005.06.009

[46] Sicard P, Falco A, Faure S, Thireau J, Lindsey SE, Chauvet N, et al. High-resolution ultrasound and speckle tracking: a non-invasive approach to assess in vivo gastrointestinal motility during development. Development. 2022;149:200625. 10.1242/dev.200625 PMC10655954 35912573

[47] Huycke TR, Miller BM, Gill HK, Nerurkar NL, Sprinzak D, Mahadevan L, et al. Genetic and mechanical regulation of intestinal smooth muscle development. Cell. 2019;179:90-105.e21. 31539501 10.1016/j.cell.2019.08.041 PMC6756183

[48] Verzi MP, Stanfel MN, Moses KA, Kim B-M, Zhang Y, Schwartz RJ, et al. Role of the homeodomain transcription factor Bapx1 in mouse distal stomach development. Gastroenterology. 2009;136:1701–10. 19208343 10.1053/j.gastro.2009.01.009 PMC2955323

[49] Degenhardt T, Saramäki A, Malinen M, Rieck M, Väisänen S, Huotari A, et al. Three members of the human pyruvate dehydrogenase kinase gene family are direct targets of the peroxisome proliferator-activated receptor beta/delta. J Mol Biol. 2007;372:341–55. 17669420 10.1016/j.jmb.2007.06.091

[50] Yoon JC, Chickering TW, Rosen ED, Dussault B, Qin Y, Soukas A, et al. Peroxisome proliferator-activated receptor gamma target gene encoding a novel angiopoietin-related protein associated with adipose differentiation. Mol Cell Biol. 2000;20:5343–9. 10866690 10.1128/mcb.20.14.5343-5349.2000 PMC85983

[51] Thoudam T, Ha C-M, Leem J, Chanda D, Park J-S, Kim H-J, et al. PDK4 augments ER-mitochondria contact to dampen skeletal muscle insulin signaling during obesity. Diabetes. 2019;68:571–86. 30523025 10.2337/db18-0363 PMC6385748

[52] McAinch AJ, Cornall LM, Watts R, Hryciw DH, O’Brien PE, Cameron-Smith D. Increased pyruvate dehydrogenase kinase expression in cultured myotubes from obese and diabetic individuals. Eur J Nutr. 2015;54:1033–43. 25311062 10.1007/s00394-014-0780-2

[53] Barres R, Kirchner H, Rasmussen M, Yan J, Kantor FR, Krook A, et al. Weight loss after gastric bypass surgery in human obesity remodels promoter methylation. Cell Rep. 2013;3:1020–7. 23583180 10.1016/j.celrep.2013.03.018

[54] Kersten S. Role and mechanism of the action of angiopoietin-like protein ANGPTL4 in plasma lipid metabolism. J Lipid Res. 2021;62: 100150. 34801488 10.1016/j.jlr.2021.100150 PMC8666355

[55] Romeo S, Pennacchio LA, Fu Y, Boerwinkle E, Tybjaerg-Hansen A, Hobbs HH, et al. Population-based resequencing of ANGPTL4 uncovers variations that reduce triglycerides and increase HDL. Nat Genet. 2007;39:513–6. 17322881 10.1038/ng1984 PMC2762948

[56] Dewey FE, Gusarova V, O’Dushlaine C, Gottesman O, Trejos J, Hunt C, et al. Inactivating variants in ANGPTL4 and risk of coronary artery disease. N Engl J Med. 2016;374:1123–33. 26933753 10.1056/NEJMoa1510926 PMC4900689

[57] Gusarova V, O’Dushlaine C, Teslovich TM, Benotti PN, Mirshahi T, Gottesman O, et al. Genetic inactivation of ANGPTL4 improves glucose homeostasis and is associated with reduced risk of diabetes. Nat Commun. 2018;9:2252. 29899519 10.1038/s41467-018-04611-z PMC5997992

