# Supplementary material for: Obesity induces phenotypic switching of gastric smooth muscle cells through the activation of the PPARD/PDK4/ANGPTL4 pathway
Source: J Biomed Sci. 2025 Jul 12;32:67. doi: 10.1186/s12929-025-01163-5 (PMC12254972; doi:10.1186/s12929-025-01163-5)
Supplement: Supplementary file 1 — Additional file 1 [file 12929_2025_1163_MOESM1_ESM.pdf]

Table S1. Clinical characteristics of patients with obesity. Data are presented as mean ±SD.

Abbreviations: BMI, body mass index; HbA1c, hemoglobin A1c; nd, not determined.

	Patients without obesity (Controls)	Patients with obesity	Patients with obesity and without diabetes	Patients with obesity and with diabetes
Number	4	15	6	9
Gender, %female	25	60	50	66.6
Age	56.5±9.1	47±11	42.3±15.4	50.6±5.9
BMI	23.4±3.3	42.2±5.8	43.4±5.8	41.5±6.1
Blood Glucose level, mmol^{-1}	nd	6.3±1.3	5.3±0.4	7±1.3
Insulinemia, mU l^{-1}	nd	15.7±9.9	12.4±3.6	17.9±12.3
C peptitde, ng ml^{-1}	nd	3.5±1.5	3±0.6	3.9±1.9
HbA1c %	nd	6.4±0.9	5.5±0.2	7±0.7

Table S2. Pathologies, surgery and treatment of control patients.

	Gender	Age	BMI	Pathologies	Treatment	Surgery
Control#1	M	62	24.7	Cardia carninoma	Radiotherapy	Esophagectomy
Control#2	F	46	26.34	Gastroesophageal reflux and gastric fistula	n/a	Partial gastrectomy
Control#3	M	52	18.69	Squamous cell carcinoma of the middle third of the esophagus	Chemotherapy and radiotherapy	Esophagectomy
Control#4	M	66	23.72	Adenocarcinoma of the lower third of the esophagus	Chemotherapy (Paclitaxel) and radiotherapy	Esophagectomy

Table S3. Relative concentration (pg/mg of proteins) of fatty acid in human gastric SMCs treated with lipids for 3 and 7 days. Values (n=6) are expressed as mean±SEM; Statistical analysis was performed using Kruskal-Wallis test followed by Dunn's multiple comparisons test. *p<0.05 compared to the control. Abbreviations: FA: fatty acid, SAFA: saturated fatty acid, MUFA: monounsaturated fatty acid, PUFA: polyunsaturated fatty acid.

	Control - 3 days	Lipid - 3 days	Control - 7 days	Lipid - 7 days
**C16:0**	126.5±18.27	157.4±29.73	136.6±10.99	175.7±14.57
**C17:0**	4.83±0.76	4.55±0.71	5.27±0.47	6.05±0.30
**C18:0**	133.3±20.55	137.3±21.70	153.4±12.65	176.5±6.707
**C20:0**	1.01±0.17	1.04±0.16	1.30±0.15	1.39±0.04
**C23:0**	4.09±0.71	5.02±0.74	4.84±0.48	9.28±0.16 *
**C16:1 n-7**	12.05±2.05	11.63±2.45	11.26±1.37	9.032±1.05
**C18:1 n-9**	240.4±38.8	347.7±58.9	272.7±20.4	360.4±19.7
**C18:1 n-7**	82.16±13.35	74.19±11.79	97.93±7.51	86.17±3.64
**C20:1 n-9**	4.43±0.77	10.43±1.63 *	8.582±0.83	16.62±0.50 *
**C22:1 n-9**	4.17±0.81	5.16±0.75	5.97±1.09	7.05±0.55
**C16:3**	13.86±2.05	14.75±2.72	15.91±2.52	22.93±1.60
**C18:2 n-6**	5.55±0.91	6.80±1.24	5.43±0.44	6.48±0.39
**C18:3 n-3**	2.33±0.39	1.86±0.48	3.09±0.26	3.47±0.14
**C20:2 n-6**	20.06±3.35	21.40±3.23	27.72±2.42	30.10±0.85
**C20:3 n-6**	7.12±1.20	7.33±1.29	7.28±0.60	9.54±0.45
**C20:4 n-6**	48.21± 7.60	60.36±9.62	57.89±5.01	92.41±3.99 *
**C22:2 n-6**	6.39±1.87	8.40±1.18	12.02±1.28	15.87±0.21
**C22:5 n-3**	5.28±0.93	9.09±1.59	4.82±0.46	9.31±0.39 **
**C22:6 n-3**	24.74±4.03	32.44±5.31	29.15±2.87	50.28±1.93 *
**Total FA**	746.6±115.7	916.9±153.8	861.2±68.8	1089±52
**Total SAFA**	269.8±39.6	305.3±52.9	301.4±23.5	369.0±20.2
**Total MUFA**	343.2±55.2	449.1±75.4	396.4±30.4	479.3±24.1
**Total PUFA**	133.5±21.6	162.4±25.6	163.3±15.5	240.4±9.2 *

Table S4. Relative concentration (pg/mg of proteins) of ceramide and sphingomyelin in gastric human SMCs treated with lipids for 3 and 7 days. Values (n=6) are expressed as mean±SEM; Statistical analysis was performed using Kruskal-Wallis test followed by Dunn's multiple comparisons test. *p<0.05; **p<0.01 compared to the control. Abbreviations: Cer: ceramide, SM: sphyngomyelin.

	Control - 3 days	Lipid - 3 days	Control - 7 days	Lipid - 7 days
Cer 18:1/16:0	984,5±108,9	351,3±25,6 **	613,2±28,5	182,8±10,9 **
Cer 18:1/16:1	14,13±1,37	4,82±0,37 **	7,08±0,31	2,25±0,15 **
Cer 18:1/18:0	193,8±19,3	46,50±3,50 **	99,70±6,03	19,26±0,98 **
Cer 18:1/18:1	10,61±1,05	3,34±0,24 **	5,99±0,30	2,28±0,18 **
Cer 18:1/20:0	52,87±5,15	12,74±1,03 **	28,59±2,32	5,74±0,29 **
Cer 18:1/22:0	353,8±34,9	90,60±7,46 **	218,4±17,3	44,17±2,42 **
Cer 18:1/24:0	1165±98	483,4±36,1 *	1001±61	349,7±18,0 **
Cer 18:1/24:1	1125±109	460,8±33,3 **	867,7±52,8	340,6±16,0 **
Cer 18:1/26:0	50,70±5,55	13,41±1,11 *	41,54±2,10	6,02±0,31 **
Cer 18:1/26:1	56,32±5,88	15,03±1,26 *	42,81±1,97	6,76±0,32 **
SM 18:1/16:0	876,8±55,9	951,4±77,8	972,9±114,7	1062±147,7
SM 18:1/16:1	48,87±2,90	61,73±4,67	50,28±5,47	56,79±7,63
SM 18:1/18:0	88,37±6,57	97,33±6,65	96,94±9,27	103,4±9,9
SM 18:1/18:1	18,03±1,30	21,63±1,40	17,74±1,62	19,80±2,01
SM 18:1/20:0	31,77±2,18	34,09±2,46	34,85±3,79	37,03±3,77
SM 18:1/20:1	7,31±0,48	8,24±0,59	7,37±0,82	7,51±0,78
SM 18:1/22:0	127,7±8,11	138,2±10,5	144,8±17,5	135,8±18,4
SM 18:1/22:1	59,99±3,74	68,54±5,30	69,40±8,73	67,82±9,37
SM 18:1/24:0	305,4±19,6	289,2±20,9	340,6±43,1	300,7±40,6
SM 18:1/24:1	606,8±36,4	700,9±54,5	696,5±90,8	763,7±112,4
Total Cer	4006±384	1482±109 *	2926±171	959±47 **
Total SM	2185±138	2380±184	2438±295	2560±352

Table S5. siRNA used in this study for inhibition.

Target	Sense	Antisense
ANGPTL4	5’-CCA-AGC-CUG-CCC-GAA-GAA-ApTpT-3’	5’-UUU-CUU-CGG-GCA-GGC-UUG-GpTpT-3’
PDK4	5’-UCU-CCA-GAA-UUA-AAG-CUU-ApTpT-3’	5’-UAA-GCU-UUA-AUU-CUG-GAG-ApTpT-3’

Table S6. List of antibodies used in this study.

Target	Manufacture	Reference
Monoclonal rabbit anti-CALPONIN1 antibody	Abcam	Cat# ab46794, RRID:AB_2291941
Monoclonal mouse anti-CALPONIN1 antibody	Santa Cruz Biotechnology	Cat# sc-58707, RRID:AB_781770
Monoclonal mouse anti-GAPDH antibody	Sigma	Cat# G8795, RRID:AB_1078991
Polyclonal rabbit anti-gamma-SMA antibody	Mybiosource	Cat# MBS820899
Monoclonal mouse anti-alpha-SMA antibody	Santa Cruz Biotechnology	Cat# sc-32251, RRID:AB_262054
Polyclonal goat anti-SM22 antibody	Abcam	Cat# ab10135, RRID:AB_2255631
Polyclonal rabbit anti-PPAR delta antibody	Abcam	Cat# ab178866, RRID:AB_2722649
Polyclonal rabbit anti-PPAR delta antibody	Fisher Scientific	Cat# PA1823, RRID:AB_2165895
Polyclonal rabbit anti-beta III TUBULIN antibody	Abcam	Cat# ab18207, RRID:AB_444319
Monoclonal Vinculin (E1E9V) XP® Rabbit mAb	Cell Signaling	Cat#13901S, RRID: AB_2728768
Donkey anti-mouse IgG (H+L) antibody, Alexa Fluor 488	Fisher scientific	Cat#10544773, RRID:AB_141607
Donkey anti-rabbit IgG (H+L) antibody, Alexa Fluor 568	Fisher scientific	Cat#10617183, RRID:AB_2534017
Donkey anti-mouse IgG (H+L) antibody, Alexa Fluor 667	Fisher scientific	Cat#10226162, RRID:AB_162542
Donkey anti-goat IgG (H+L) Alexa Fluor® 488	Fisher scientific	Cat#15930877, RRID:AB_2762838
Goat Anti-Rabbit IgG Antibody (H+L), Biotinylated	Vector Lab	Cat#BA-1000, RRID:AB_2313606
Goat Anti-Rabbit IgG Antibody (H+L), Biotinylated	Vector Lab	Cat#BA-1000, RRID:AB_2313606
Goat Anti-Mouse IgG Antibody (H+L), Biotinylated, R.T.U.	Vector Lab	Cat#BP-9200, RRID:AB_2336171
Monoclonal mouse anti-GAPDH antibody	Sigma	Cat# G8795, RRID:AB_1078991

Footnote: *RRID = antibody register number (https://www.antibodyregistry.org/)*

Table S7. Human gene-specific primers used for RT-qPCR.

Target	Forward primer	Reverse primer	Amplicon (bp)
ACTG2	ATACCCCATTGAACACGGCAT	TTAGGGGAGCCTCTGTGAGC	128
ANGPTL4	TCTCTGGAGGCTGGTGGTTT	CCAGGTCTTCCAGAAGATTCCC	116
C-MYC	GTGGTCTTCCCCTACCCTCT	GCTGCGTAGTTGTGCTGATG	356
HMBS	GGCAATGCGGCTGCAA	GGGTACCCACGCGAATCAC	64
LIX1	CTGCAGTGACCCTGACACTT	AATCTGGCCTCTGCCATCAC	118
PDK4	AGAGGTGGAGCATTTCTCGC	ATGTTGGCGAGTCTCACAGG	138
RPLPO	TCATCCAGCAGGTGTTCG	AGCAAGTGGGAAGGTGTAA	224
PPARD	TACGAGAAGTGTGAGCGCAG	CCAGCTTCCTCTTCTCAGCC	503
YWHAZ	ACTTTTGGTACATTGTGGCTTCAA	CCGCCAGGACAAACCAGTAT	94
