# Supplementary material for: Obesity induces phenotypic switching of gastric smooth muscle cells through the activation of the PPARD/PDK4/ANGPTL4 pathway
Source: J Biomed Sci. 2025 Jul 12;32:67. doi: 10.1186/s12929-025-01163-5 (PMC12254972; doi:10.1186/s12929-025-01163-5)
Supplement: Supplementary file 2 — Additional file 2 [file 12929_2025_1163_MOESM2_ESM.pdf]

Supplemental Fig. 1 Impact of HDF containing 60% fat (230-HFD) for 12 weeks on metabolic status of male adult mice. Comparison of control water group to HFD group mice demonstrated global metabolic alteration with increase of body weight (A), adipocyte index (B), TA % (C), Fasting blood glucose (D), Total Cholesterol (E) and LDL (F). On similar way, HFD group mice present elevated oral glucose tolerance test (G) and Total caloric intake (H) compare to control water group mice. Data are presented as the mean ± SEM and Student's t test was applied (**$P < 0.01$; ***$P < 0.001$; ****$P < 0.0001$) (A-F).

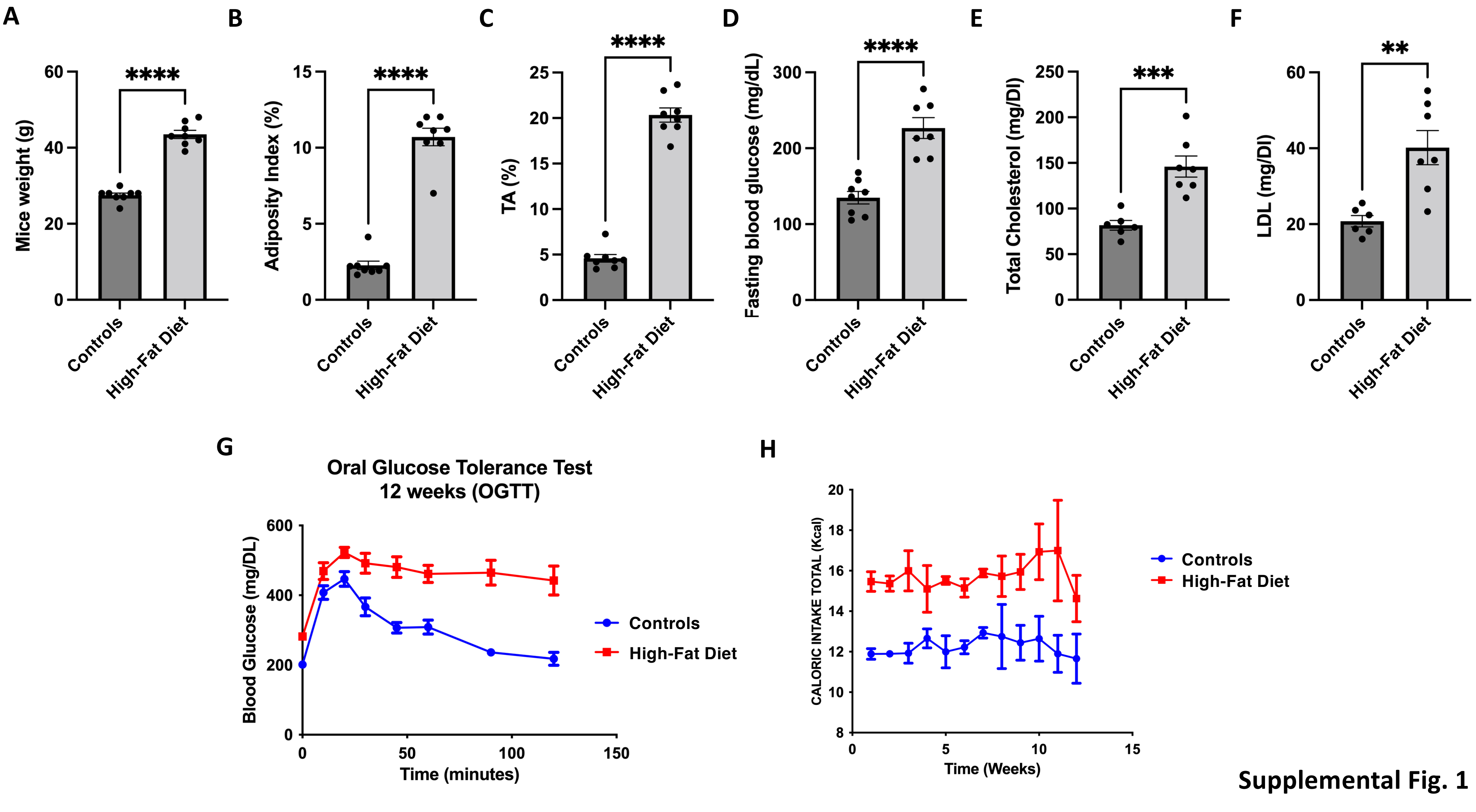

Supplemental Fig. 1

Supplemental Fig. 2 (A) Venn diagram with the number of genes up-regulated and down-regulated using a significance cutoff of $P<0.01$ at 3 days (upper panel) and 7 days (lower panel) post-lipid treatment compared to their respective controls. (B) Diagrams representing the individual expression of *ANGPTL4* (left panel) and *PDK4* (right panel) identified in human gastric SMC cultures with or without lipid treatment for 3 days. (C) Diagrams representing the individual expression of *ANGPTL4* (left panel) and *PDK4* (right panel) identified by RNA sequencing in human gastric SMC cultures with or without lipid treatment for 7 days.

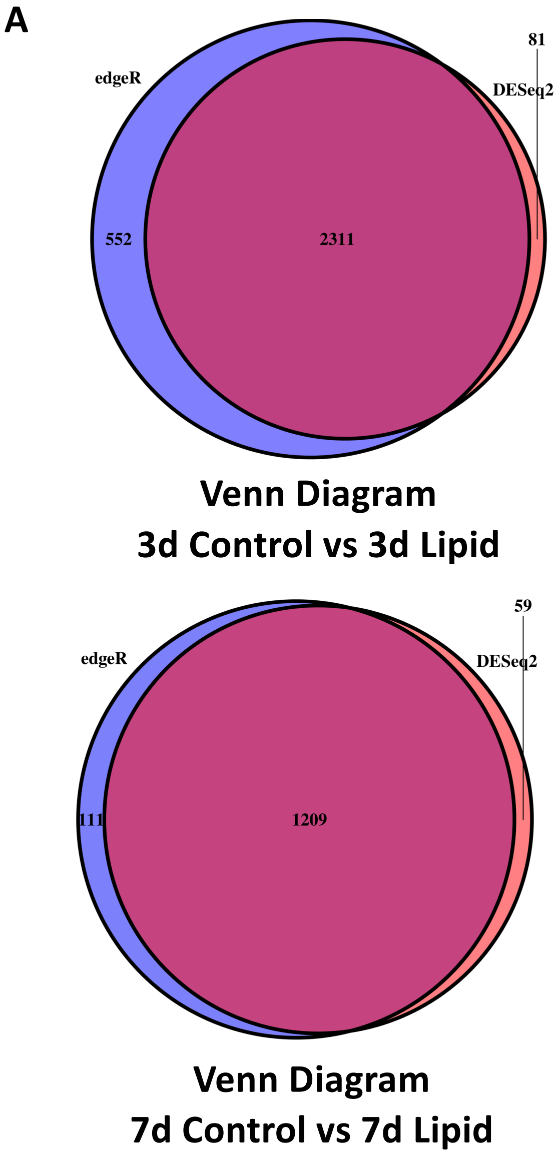

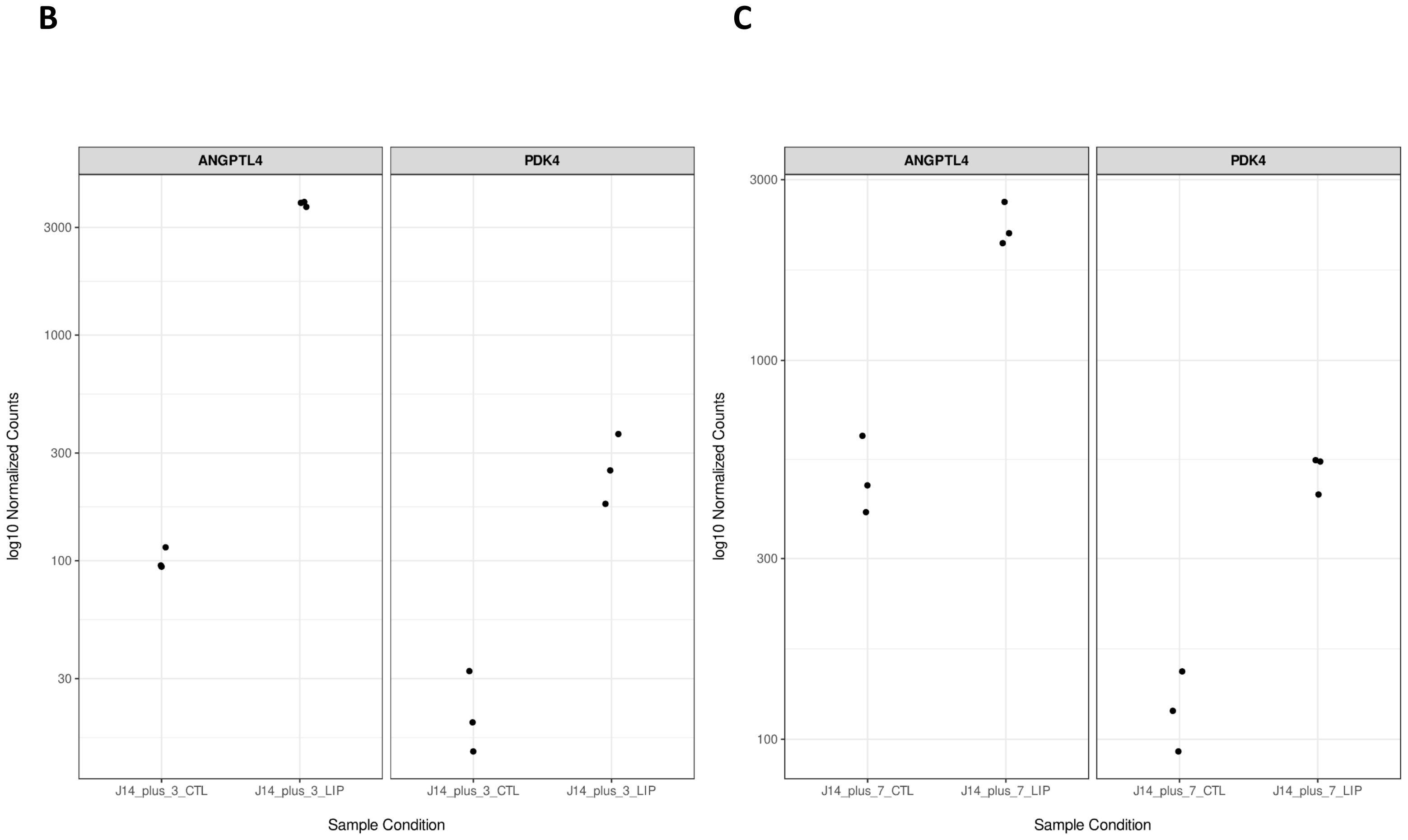

Supplemental Fig. 2

Supplemental Fig. 3 Relative mRNA expression of inflammatory genes identified by RNA sequencing using a significance cutoff of $P<0.01$ in human gastric SMC cultures with or without lipid treatment for 3 and 7 days. Average of n=3.

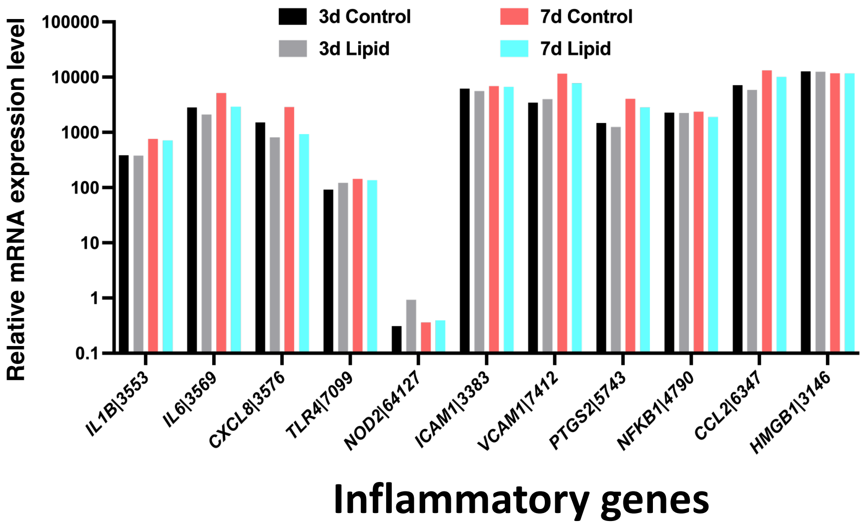

Supplemental Fig. 3

Supplemental Fig. 4 Characterization of WRAP5:siRNA nanoparticles used in this study. All WRAP5:siRNA complexes were formed at room temperature using a molar ratio (R) of 20 (WRAP5:siRNA=20:1). (A) Examples of the size distribution of si-*PDK4*, si-*ANGPTL4* and si-*NEG* formulated with WRAP5 and determined by DLS technology. (B) The table indicated the mean size (Z-Ave) and particle distribution of homogeneity (Pdl) of each nanoparticles used. (C) Graphical representation of the relative cytotoxicity (%) as measured using an LDH assay after transfection with WRAP5:si-*NEG* complexes on human gastric SMCs. Non-treated cells were used as negative control (0% toxicity) whereas Triton-treated cells were used as negative control (100% toxicity). Cytotoxic condition (~20%) is not encountered in all used siRNA concentrations excepts in triton induced-cytotoxic condition (10%).

A

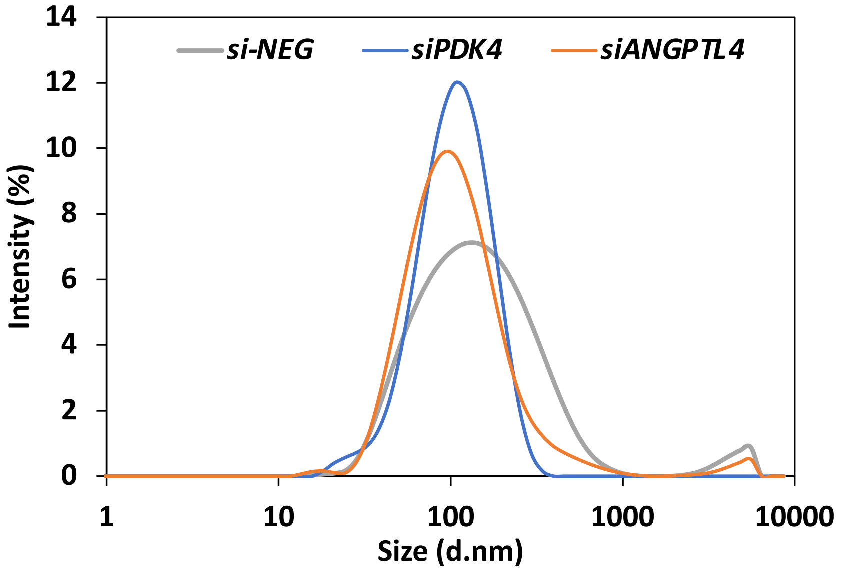

B

Sample Name	Z-Ave (d.nm)	PdI
si-*NEG*	121.9	0.393
si-*PDK4*	93.6	0.224
si-*ANGPTL4*	96.4	0.317

C

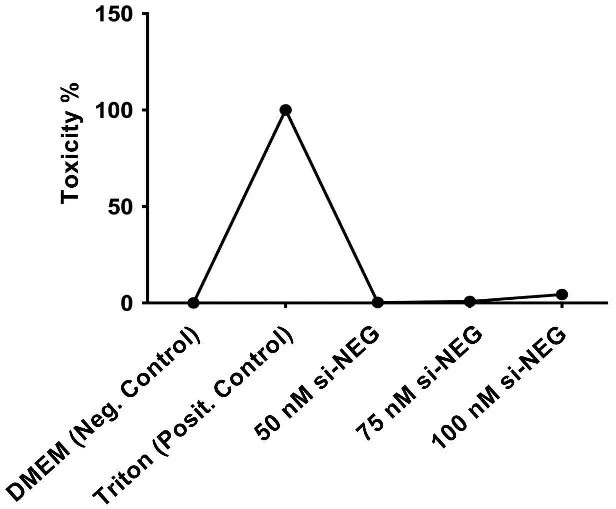

Supplemental Fig. 4

Supplemental Fig. 5 Evaluation of long-term consequences of lipid treatment in human gastric SMCs. (A) Representative Western blot of human gastric SMC extracts after 14 days of culture, with or without lipid treatment for an additional 7 or 14 days (without renewal medium) probed with antibodies directed against specific smooth muscle proteins (SM22, CALPONIN1, and γSMA) and against GAPDH as loading control. (B) Quantification of Western blot assays (n=4) comparing extracts from lipid-treated SMCs to extracts from control SMCs after 7 and 14 days of treatment. Data are presented as the mean ± SEM and two-way ANOVA test was applied (*P<0.05; **P<0.01; ****P<0.0001; ns>0.05). Lipid exposure led to a reduction in CALPONIN1 and SM22 markers at each time point. Compared to controls, CALPONIN1 expression decreased by 49% at day 7 and 48% at day 14, while SM22 expression decreased by 66% at day 7 and 54% at day 14. These results indicate a similar degree of dedifferentiation despite the prolonged treatment duration, although CALPONIN1 levels remained slightly higher at day 14. (C) RT-qPCR of *PDK4* (left panel) and *ANGPTL4* (right panel) relative mRNA level of human gastric SMC cultures after 14 days of culture, with or without lipid treatment for an additional 7 or 14 days (without renewal medium). Data were normalized to the house-keeping *HMBS* expression. Values are presented as the mean ± SEM of n=4 samples and two-way ANOVA test was applied (*P<0.05; **P<0.01; ***P<0.001; ns>0.05). Lipid exposure led to an upregulation of *PDK4* and *ANGPTL4* expression at all time point. Compared to controls, *PDK4* expression increased by 158% at day 7 and 271% at day 14, whereas *ANGPTL4* expression increased by 209% at day 7 and 276% at day 14. These findings indicate a similar comparable induction of *ANGPTL4*, but a markedly greater upregulation of *PDK4* over time.

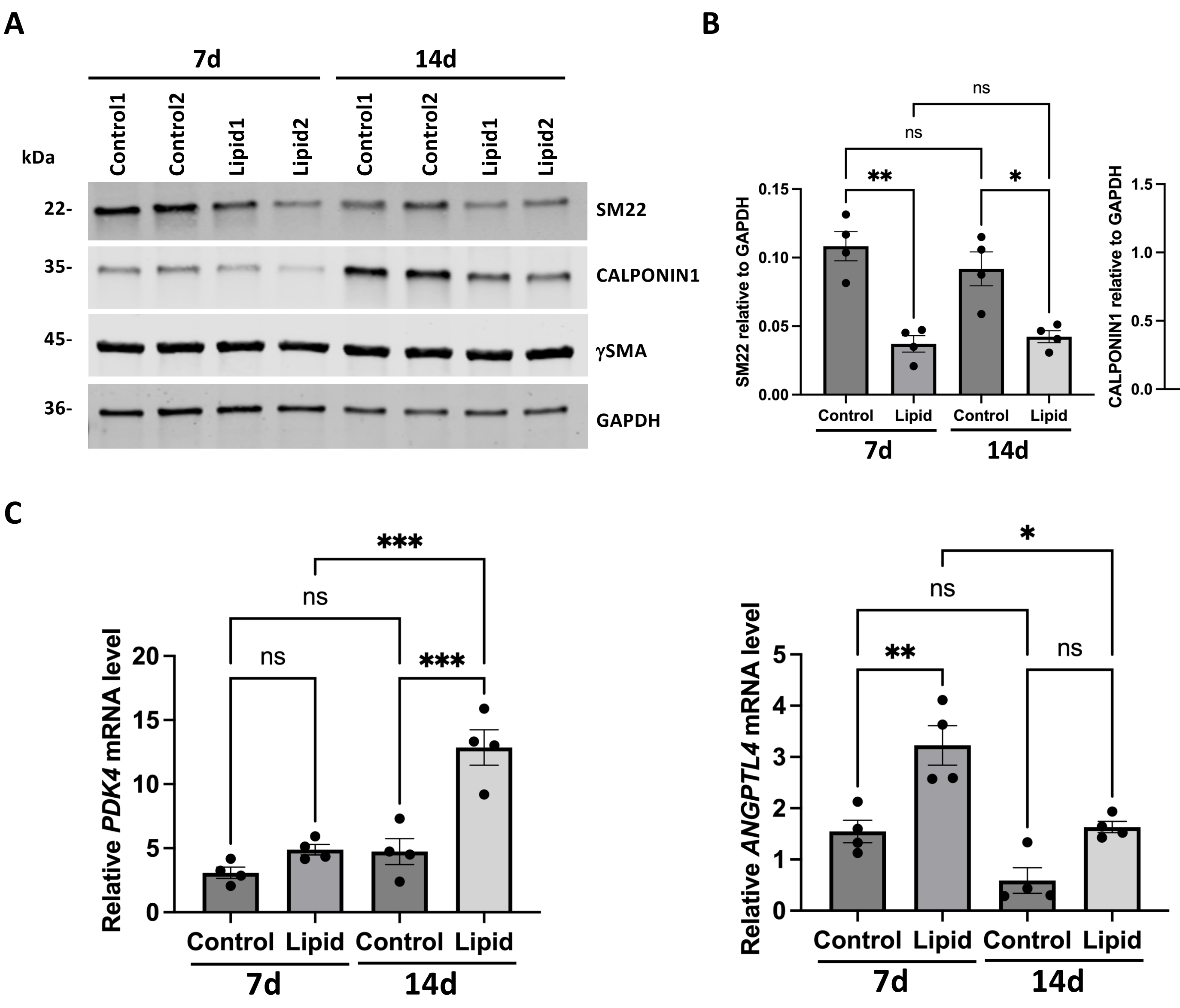

Supplemental Fig. 5

Supplemental Fig. 6 Evaluation of recovery capacity of human gastric SMC cultures following lipid-induced dedifferentiation. (A) RT-qPCR of *PDK4* (left panel) and *ANGPTL4* (right panel) relative mRNA level in human gastric SMC cultures after 14 days of culture, with or without lipid treatment for an additional 7 days and with or without lipid treatment for an additional 7 days (with renewal medium for Control, Lipid and LipidWash (removing lipid after 7 days) conditions)**.** Data were normalized to the house-keeping *HMBS* expression. Values are presented as the mean ± SEM of n=4 samples. Two-way ANOVA test was applied for each time and condition (*P<0.05; ***P<0.001; ****P<0.0001; ns>0.05). Two-way ANOVA revealed that the stimulation of *PDK4* and *ANGPTL4* mRNA levels returned to baseline after lipid removal, becoming statistically indistinguishable from untreated controls. (B) Representative Western blot of human gastric SMC extracts after 14 days of culture, with or without lipid treatment for an additional 7 days and with or without lipid treatment for an additional 7 days (with renewal medium for Control, Lipid and LipidWash (removing lipid after 7 days) conditions) than probed with antibodies directed against specific smooth muscle proteins (SM22, CALPONIN1, and γSMA) and against GAPDH as loading control. (C) Quantification of Western blot assays (n=4) comparing extracts from lipid-treated SMCs to extracts from control SMCs at 7 days and 14 days including the LipidWash condition. Data are presented as the mean ± SEM and two-way ANOVA test was applied (*P<0.05; **P<0.01; ns>0.05). Two-way ANOVA analysis revealed that removal of the lipid medium led to a statistically significant increase of SM22 expression level, whereas CALPONIN1 expression remained low.

Supplemental Fig. 6

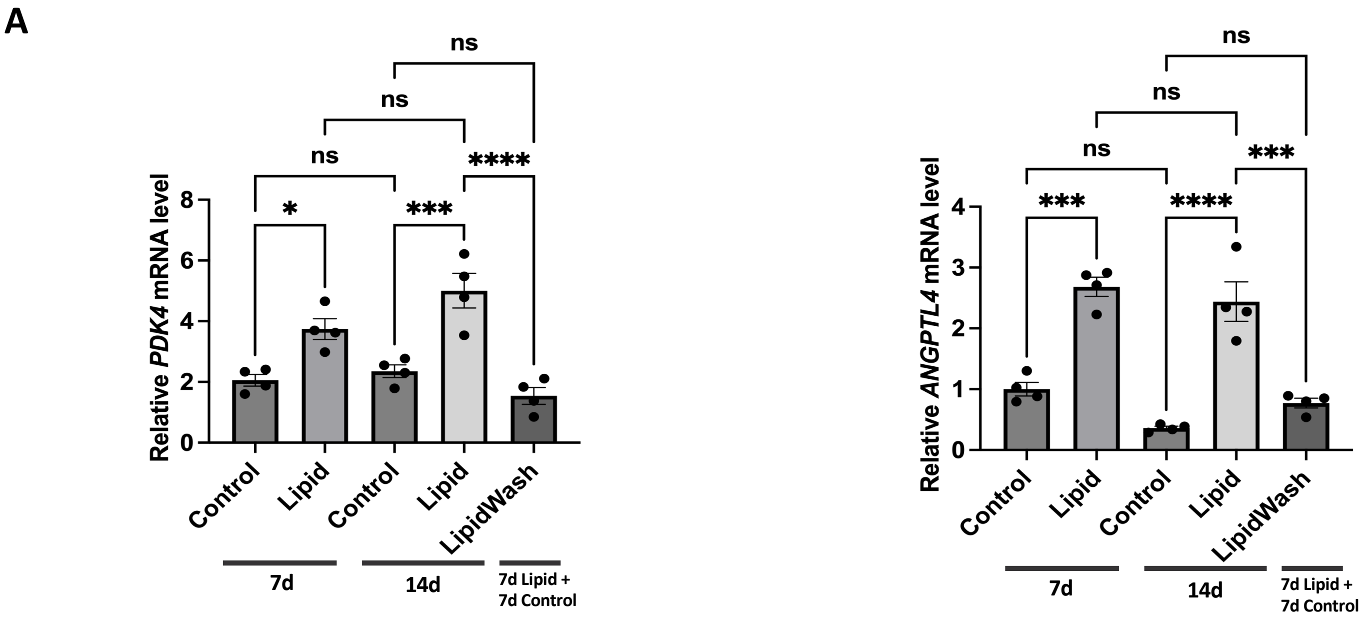

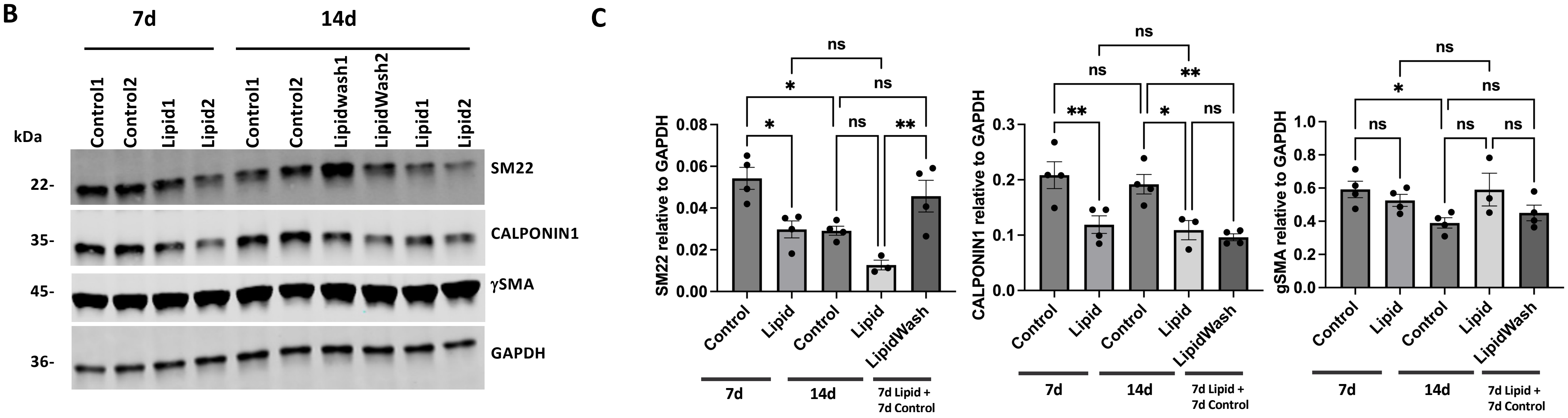

Supplemental Fig. 7 Evaluation of mRNA stability of *PDK4* and *ANGPTL4* after lipid treatment on human gastric SMC cultures. RT-qPCR of *PDK4*, *ANGPTL4, C-MYC and ACTG2* relative mRNA fold-level in human gastric SMC cultures with or without lipid treatment for 3 days followed by actinomycin-D treatment for 30 minutes, 1, 2, 4 and 6 hours. Data were normalized to the house-keeping *RPLPO* expression (house-keeping gene stable over 6 hours of actinomycin treatment) and to levels of target gene at 0 hour of actinomycin-D treatment. *C-MYC* was used as control of unstable transcript and *ACTG2* as control of stable transcript. Values are presented as the mean ± SEM of n=4 samples. Simple linear regression test (A) and Mixed effect analysis for multiple comparisons (B) (with *P<0.05; ns>0.05) were applied for each time and condition. *PDK4* and *ANGTPL4* mRNA profiles revealed no significant differences in half-life or decay kinetics between the control and lipid-treated conditions.

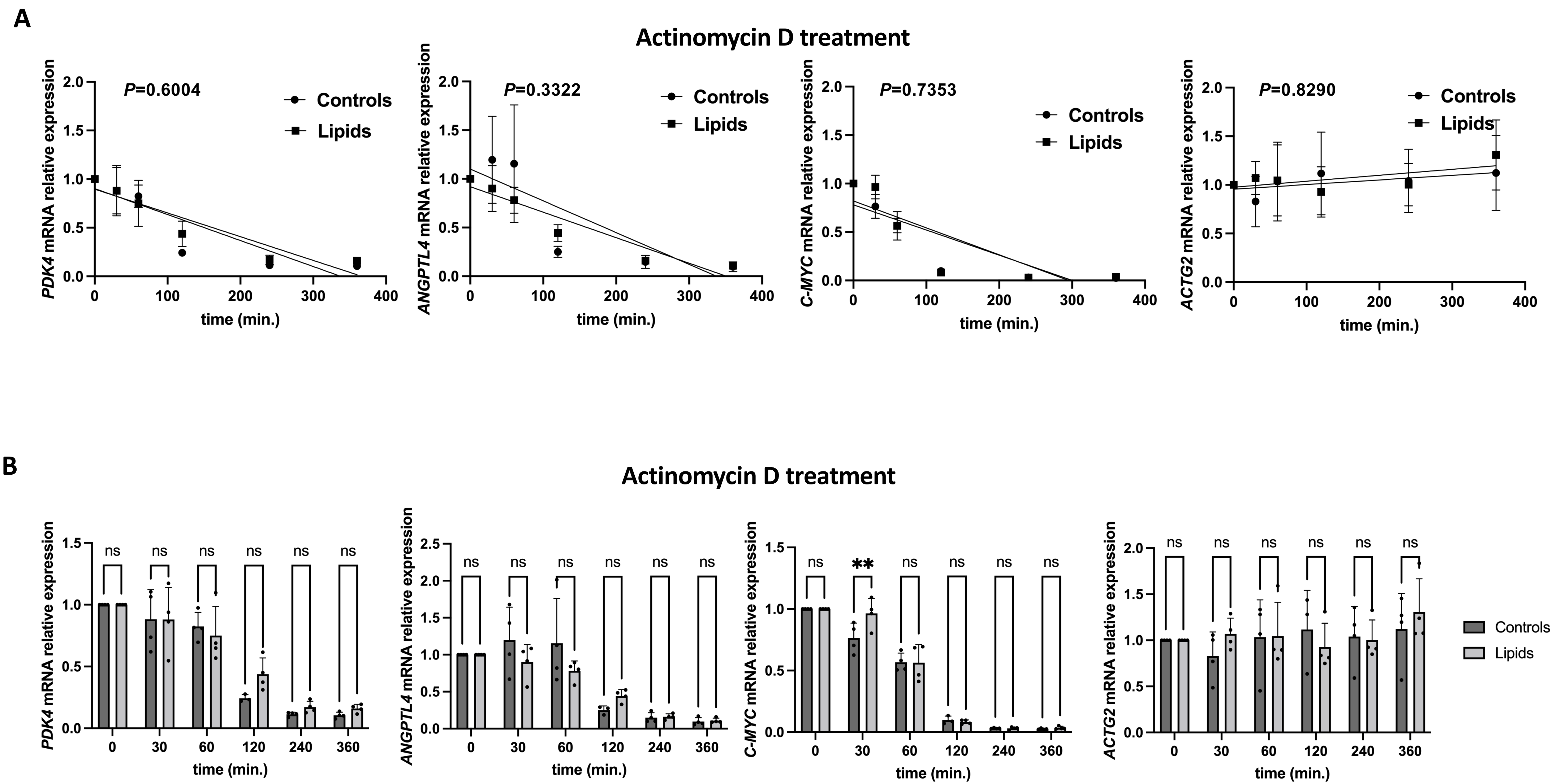

Supplemental Fig. 7

Supplemental Fig. 8 (A) mRNA expression of PPARA, PPARD and PPARG determinates by RNA sequencing from human gastric SMC cultures with or without lipid treatment for 3 and 7 days. Average of n=3. PPARD is strongly present in all conditions. (B) Promoter analyses of vertebrate *PDK4* (upper panel) and *ANGPTL4* (lower panel) genes using Contrav3 identified one PPAR-RXR binding site highly conserved across vertebrate species for *PDK4* and one exclusively conserved in primates for *ANGPTL4*.

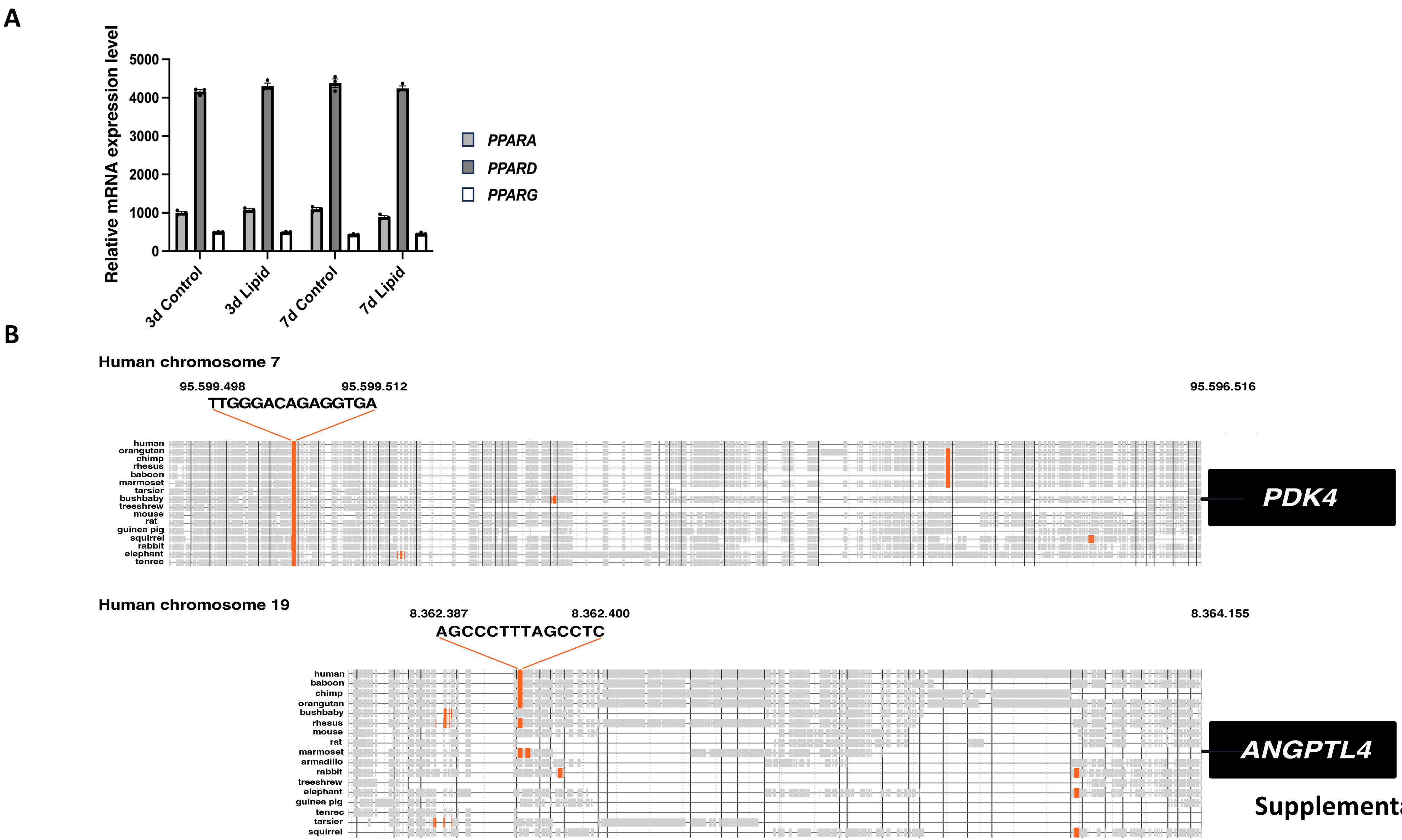

Supplemental Fig. 8

Supplemental Fig. 9 Impact of GSK3787, covalent antagonist of PPARD, on the lipid-inducted SMC dedifferentiation process. (A) RT-qPCR of *PDK4* (left panel) and *ANGPTL4* (right panel) relative mRNA levels in human gastric SMC cultures treated for 3 days with GSK3787 (PPARD antagonist, 5 μM), with lipid, and with lipid+GSK3787 compared to untreated SMC (Control). Data were normalized to the house-keeping *HMBS* expression. Values are the mean ± SEM of n=3 samples. ****P0.05 and ns>0.05 (One way ANOVA, multiple comparison test). PDK4 and ANGPTL4 stimulation induced by lipid treatment were significantly reduced when combined with GSK3787, approaching control level. (B) Western blot of human gastric SMC extracts from 14 days of culture treated for 3 days with GSK3787 (PPARD antagonist, 5 μM), with lipid, and with lipid+GSK3787 compared to untreated SMC (Control) and probed with antibodies directed against SM22, CALPONIN1, and γSMA and GAPDH as loading control. (C) Quantification of Western blot analyses (n=4) comparing protein extracts from SMCs treated with lipids or with lipids+GSK3787 to those from control SMCs. SM22 expression level in lipids+GSK3787 condition was statistically similar to those in control cells, whereas CALPONIN1 expression remained reduced.

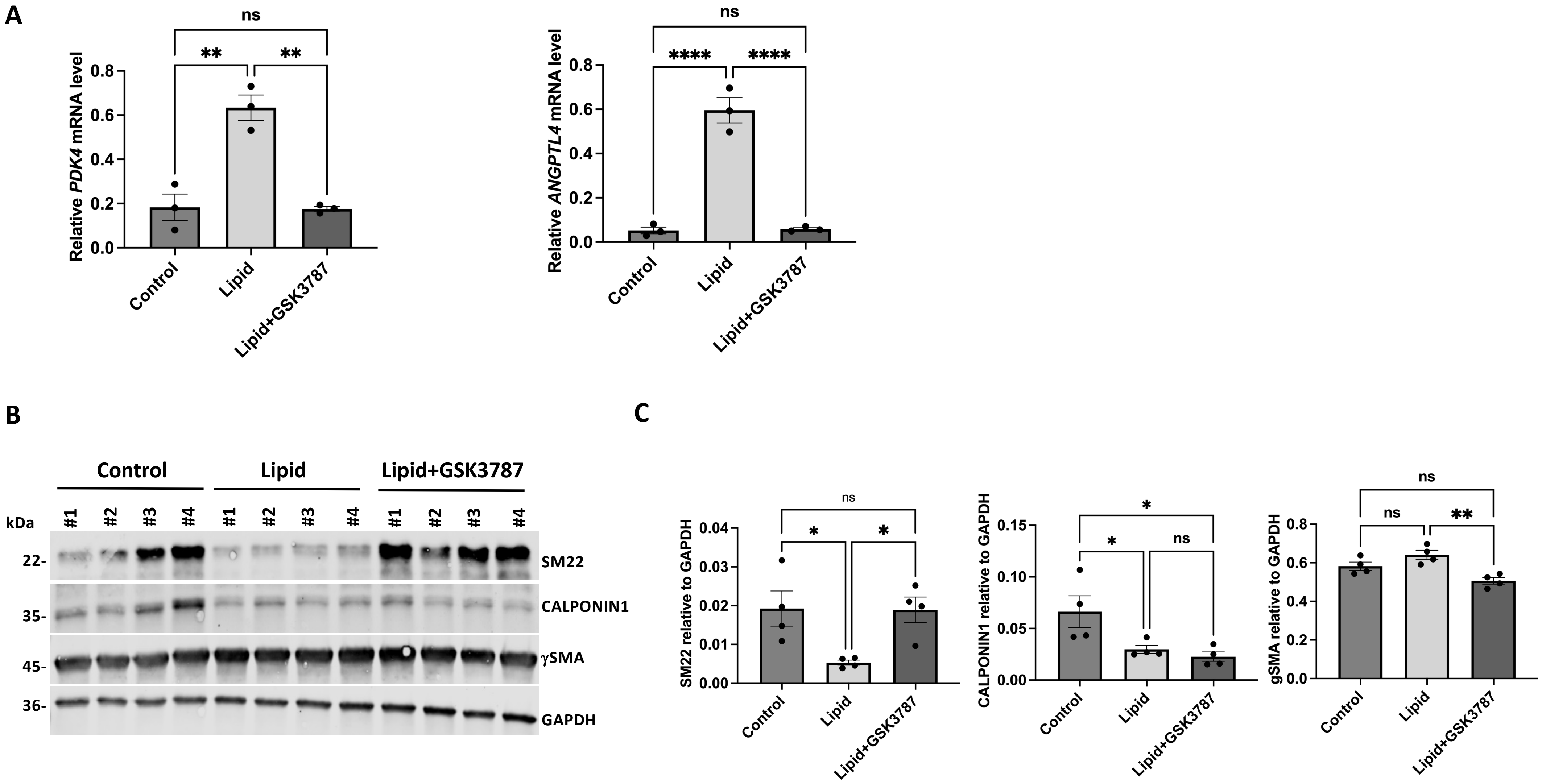

Supplemental Fig. 9
